# Esterification of Lutein from Japanese Knotweed Waste Gives a Range of Lutein Diester Products with Unique Chemical Stability

**DOI:** 10.1021/acssuschemeng.2c01241

**Published:** 2022-04-28

**Authors:** Valentina Metličar, Alen Albreht

**Affiliations:** † Laboratory for Food Chemistry, Department of Analytical Chemistry, National Institute of Chemistry, Hajdrihova 19, Ljubljana SI-1000, Slovenia; ‡ Faculty of Chemistry and Chemical Technology, University of Ljubljana, Večna pot 113, Ljubljana SI-1000, Slovenia

**Keywords:** xanthophylls, lutein, capsanthin, β-cryptoxanthin, zeaxanthin, violaxanthin, supercritical CO_2_ extraction, invasive alien plant species

## Abstract

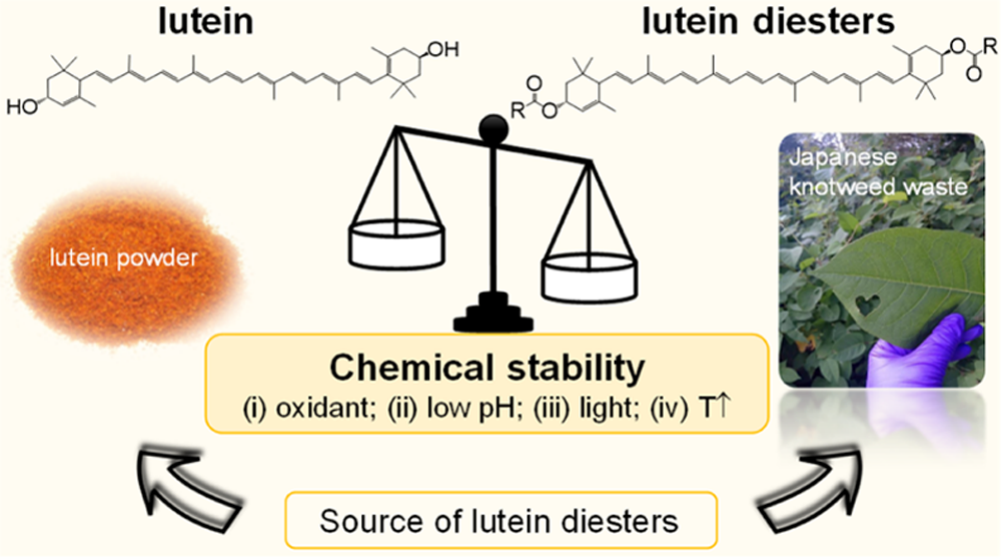

A valorization strategy for an aggravating type of plant waste is put to the test herein. It envisions the use of Japanese knotweed green leaves as a sustainable source of free lutein, from which bioactive diesters could be prepared as potential value-added products with improved properties. To this end, 13 structurally distinct model lutein diesters were synthesized and the relationships between their structure and stability were systematically determined. The forced degradation data show that the stability of a particular lutein diester may depend to a large extent on the type of exposure (elevated temperature, light, oxidant, or acidic environment) and, more importantly, not every esterification attempt necessarily leads to an enhancement of lutein’s chemical stability. However, three branched and bulky products—lutein di(2,2-dimethylpropanoate), lutein di(2-methylpropanoate), and lutein di(3-methylbutanoate)—proved to be particularly relevant, as they consistently exhibited 1.5–21-fold higher stability compared to free lutein, regardless of the stress conditions used. Finally, we show that the Japanese knotweed plant matrix had a significant negative or positive effect on pigment degradation kinetics that could not be easily predicted. Thus, the proposed valorization strategy is quite feasible, but the esterification approach should be tailored to the intended use of a lutein diester.

## Abstract 2

Potential value-added products were prepared through rational design by using waste of Japanese knotweed for the production of lutein diesters with enhanced chemical stability.

## Introduction

Japanese knotweed (*Fallopia japonica* Houtt), giant knotweed (*Fallopia sachalinensis* F.), and their hybrid—Bohemian knotweed (*Fallopia* × *bohemica*)—are members of the *Polygonaceae* family.^1^ Although native to eastern Asia, they are considered invasive alien plant species in 42 U.S. states, throughout Europe, New Zealand, and Australia, threatening local biodiversity and environmental sustainability and causing enormous economic damage. In particular, Japanese knotweed has been identified by the International Union for Conservation of Nature as one of the 100 most invasive alien plant species.^2^ It is highly resistant to any kind of eradication strategy (biological, chemical, or mechanical), but plant excavation or plant harvest with its subsequent incineration seems to be the most sensible way of controlling it at present.^3^ Nonetheless, due to a high energy demand and a large carbon footprint, this particular approach to waste management is not sustainable in the long term. Therefore, different valorization strategies for this plant are now actively being explored. As one of the most affected areas in the world, the UK records an infestation of Japanese knotweed for every 4 square miles, and with its biomass yield reaching a staggering 15.9 tons per hectare, this invasive alien plant species represents a virtually unlimited natural resource.^4,5^ Through innovation, the Japanese knotweed (waste) material could be exploited to generate a variety of value-added products because, despite its negative connotations, it is a rich source of several groups of bioactive compounds.^6−9^ One of these groups is also represented by photosynthetic xanthophylls, which are found primarily in the green leaves, where they regulate energy dissipation during photosynthesis.^10^ Xanthophylls are oxygen-containing carotenoids that represent an important group of secondary metabolites with various health-promoting effects such as antioxidant, anticancer, anti-inflammatory, antidiabetic, neurological, and cardiovascular protective effects.^11,12^ For instance, lutein and zeaxanthin play a key role in preventing age-related macular degeneration,^13^ while β-cryptoxanthin is a vitamin A precursor and prevents bone loss.^14^ However, there is one major issue. All-*trans* xanthophylls are rapidly degraded or isomerized into one of their *cis* isomers when isolated from plant tissues and unintentionally exposed to light, elevated temperatures, oxidants, acids, or metal ions.^15^ This genuine sensitivity not only of xanthophylls but also of carotenoids, in general, has sparked numerous research studies, and consequently there is now a whole arsenal of mature technologies that can be used for their stabilization, such as encapsulation in various protective shell materials, emulsification, complexation with macromolecules, and micelle and liposome formation.^16−18^ There is accumulating evidence that the chemical stability of xanthophylls bearing at least one hydroxyl group could also be improved by esterification with various carboxylic acids, an approach that offers multiple advantages.^17^ Recently, green leaves of Japanese knotweed have been used as a source of lutein to produce a structurally diverse series of lutein diesters with potentially improved physicochemical properties such as chemical stability.^19^ However, can the improvement be generally attributed to any given lutein (or xanthophyll) ester and under any given condition?

Systematic and comprehensive studies that could provide an answer and support a future rational design of bioactive xanthophylls from waste materials are rather scarce. Most of the available literature reports on the stability of endogenous esterified pigments in (processed) foods and food supplements or compares the relative stability of a limited number of synthesized model xanthophyll esters in solution.^20−27^ Conclusions drawn from independent studies can therefore be inconsistent or the data may not be directly comparable because these studies use different methods, experimental designs, and different model xanthophylls and matrices. For instance, unannotated lutein, β-cryptoxanthin, and zeaxanthin esters from blood oranges were shown to resist thermal degradation better than their free forms, whereas the opposite was observed for the epoxy xanthophylls, violaxanthin and antheraxanthin.^28^ Increased thermal stability was also demonstrated for β-cryptoxanthin palmitate, laurate, and myristate (synthesized from the saponified mandarin extract),^29^ but, on the other hand, β-cryptoxanthin palmitate embedded in liposomes was found to be more sensitive to UVA irradiation than free β-cryptoxanthin.^26^ The above exemplary disagreements warrant further and deeper investigation into the relationship between the structure of a particular xanthophyll ester and its chemical stability.

Therefore, the main aim of this study was to investigate whether lutein diesters produced from the waste material of Japanese knotweed leaves indeed demonstrate the anticipated increased chemical stability that would render potential value-added products in practice. In search of an answer, a number of unresolved but important subquestions emerged: (i) Can a particular lutein esterification strategy lead to its increased stability? (ii) Against which stress factor(s) does esterification actually protect the pigment? (iii) Do certain matrix components of Japanese knotweed leaves influence the stability of an esterified xanthophyll? If so, (iv) what is the direction and magnitude of the effect and is it characteristic for the whole group of xanthophyll esters or is it case-specific? To answer all these questions, we prepared 13 lutein diesters from either pure (commercially available) lutein or lutein extracted from Japanese knotweed leaves and then systematically investigated their resistance to light, elevated temperatures, oxidants, and acids, as part of a comprehensive forced degradation study. The results of this work represent an important step toward a functional valorization strategy for the waste produced in large quantities during mechanical control of this highly invasive alien plant species.

## Experimental Section

All chemicals and materials used in this study are listed in the Supporting Information.

### Carotenoid Extraction from Japanese Knotweed Green Leaves

Green leaves of Japanese knotweed were harvested in the Polje region of Ljubljana in September 2019. These were made clean of any dirt or particulate matter, air dried, and finally pulverized. Afterward, carotenoids were extracted from the processed plant material by supercritical CO_2_ (sc-CO_2_) extraction as described previously.^19^ To summarize, 5 kg of dried pulverized plant material was loaded into an extraction cell and was then extracted with sc-CO_2_ for 24 h at 150 bar, 65 °C, and at a flow rate of 120 g CO_2_/min. The extract, taking the form of a viscous paste, was being collected in a separator held at 80 bar and 45 °C. Extract solutions were afterward prepared by dissolving an aliquot (100 mg) of the sc-CO_2_ extract (total extract yield was 66 g) in ethyl acetate (EtOAc) (10 mL). These solutions were then filtered through a 0.2 μm polyvinylidene fluoride membrane (LLG labware, Meckenheim, Germany) and stored in amber glass vials (National Scientific Company, USA) at −80 °C prior to use.

### Synthesis and Purification of Lutein Diesters

For the synthesis and purification of lutein diesters, a recently developed green procedure was followed with minor modifications.^19^ Briefly, ethanolic standard solution of lutein (0.5 nM; 500 μL) was transferred into a reaction vessel (an amber 4 mL storage vial), the solvent was evaporated under a stream of argon, and the solid residue was redissolved in EtOAc (250 μL). Then, EtOAc solutions of a corresponding acid anhydride (37.5 nM; 200 μL) and 4-dimethylaminopyridine (DMAP) (75 nM; 200 μL) were added at a molar stoichiometric ratio of lutein/acid anhydride/DMAP = 1:30:60. For the reactions carried out on the crude sc-CO_2_ extract of Japanese knotweed leaves, the molar stoichiometric ratio of lutein/acid anhydride/DMAP was 1:150:200 to compensate for the loss of reagent, consumed by the interfering compounds from the plant matrix. In the case of 3-methylbutyric, 2-methylpropionic, and 2,2-dimethylpropionic anhydrides, the molar stoichiometric ratio used was 1:500:500 in order to efficiently drive the reaction forward. The reaction mixtures (approximately 650 μL in total) were stirred at ambient temperature (22 °C) in an inert argon atmosphere and in the absence of light for 24 h. Individual lutein diesters, irrespective of the source of the free lutein, were afterward purified from the reaction mixture using solid-phase extraction (SPE) on C18 SPE cartridges (3 CC/200 mg; Varian, Harbor City, USA). SPE cartridges were first conditioned with acetone (3 mL), equilibrated with an isopropanol/EtOAc/water (1:1:1; v/v/v) mixture (3 mL), then the reaction mixture (1 mL), diluted 10-fold with 85% EtOH_(aq)_ beforehand (95% EtOH_(aq)_ for lutein dipalmitate and lutein dioleate and 60% EtOH_(aq)_ for lutein diphthalate), and was loaded onto the cartridge that was further washed with 80% EtOH_(aq)_ (30% EtOH_(aq)_ in the case of lutein diphthalate and 0.5% NH_3_ in 95% EtOH_(aq)_ in the case of lutein dipalmitate and lutein dioleate; 3 mL). Lutein diester products were finally eluted from the SPE cartridge with acetone (2 mL). Acetone was removed under a stream of nitrogen, and the solid products were subsequently redissolved in EtOH to a working concentration of 10–19.3 mg/L [determined by high-performance liquid chromatographic (HPLC) analysis], so that the molar concentrations were identical for all studied lutein diesters.

### Forced Degradation Studies

For xanthophyll-forced degradation studies, all solutions of free xanthophylls (capsanthin, β-cryptoxanthin, lutein, zeaxanthin, and violaxanthin) and all 13 synthesized lutein diesters (lutein diacetate, dipropanoate, di(2,2-dimethylpropanoate), di(2-methylpropanoate), di(3-methylbutanoate), divalerate, di(pent-4-enoate), dibenzoate, didecanoate, dipalmitate, dioleate, di(pentafluoropropanoate), and diphthalate), either pure or within the Japanese knotweed leaf extract matrix, were prepared at an equimolar concentration level (approximately 20 μM in EtOH). Prepared xanthophyll solutions were individually exposed to (i) an increased temperature, (ii) UV illumination, (iii) an oxidant, and (iv) an acidic environment. For an easier grasp of the entire experimental setup, the reader is referred to Scheme S1.

Exact conditions for each forced degradation experiment were chosen based on preliminary tests carried out on free lutein, which resulted in good experimental repeatability and compound retention of about 2–33% after 7 days (see Supporting Discussion 1). All test solutions for the forced degradation studies were prepared in triplicate. In each experiment, the individual xanthophyll-containing solution was divided equally into five identical and tagged vessels and then exposed to the chosen stress condition. The solutions were sampled at the specified time intervals (0, 1, 2, 4, and 7 days) by removing the appropriate sample vessel each time at the same time of the day and storing it immediately at −80 °C until all samples from the same series were collected and afterward analyzed within 24 h.

#### Temperature

Test solutions were kept in the dark in a sealed amber HPLC vial under an argon atmosphere for 7 days at 60 °C. Control solutions were exposed to the same conditions, but kept at 22 °C.

#### Light

Test solutions were kept at 22 °C for 7 days in sealed clear borosilicate glass HPLC vials under an argon atmosphere and placed 15 cm from the UV light source [Sylvania 8W blacklight blue lamp (F8 T5 BLB 8W) with spectral peak maximum at 365 nm and ≥10 UV-A irradiance at 1 m μW/cm^2^]. Control solutions were exposed to the same conditions, but amber glass HPLC vials, which were wrapped in aluminum foil, were used instead.

#### Oxidant

Test solutions, consisting of an ethanolic solution of an individual studied xanthophyll and 3% H_2_O_2(aq)_ (95:5, v/v; total 0.15% H_2_O_2_ in final solutions), were kept in the dark in a sealed amber HPLC vial under an argon atmosphere for 7 days at 22 °C. Control solutions were prepared by replacing the peroxide solution with water.

#### Acidic Environment

Test solutions, consisting of an ethanolic solution of an individual studied xanthophyll and 200 mM ammonium formate buffer adjusted to pH = 2 (95:5, v/v; total 10 mM buffer in final solutions), were kept in the dark in a sealed amber HPLC vial under an argon atmosphere for 7 days at 22 °C. Control solutions were prepared by replacing the buffered solution with water.

### Chromatographic Analyses

Chromatographic systems and conditions for HPLC–PDA–MS^2^ analysis of free and esterified xanthophylls are given in the Supporting Information, along with the conditions for the HPLC–PDA analysis of acid anhydrides and their corresponding carboxylic acids and DMAP.

## Results and Discussion

There is a lack of uniform information on how the selection of a carboxylic acid used to produce a particular xanthophyll ester in a particular environment (e.g., solution, plant, and food) affects the chemical stability of the parent compound. Thus, we systematically evaluated the degree of degradation of 13 structurally distinct lutein diesters after independently exposing them to four stress conditions (elevated temperature, light, oxidant, and an acidic environment). The model lutein diesters were synthesized from both commercial lutein and lutein extracted from Japanese knotweed leaves. Differences in the stability data between the two subgroups were determined to reveal whether and how this ubiquitous plant waste material can be effectively used in practice for the preparation of value-added products. To ensure a reliable evaluation of the (de)stabilizing effect of a particular lutein esterification strategy, we first established a robust and appropriate methodological framework for the forced degradation experiments. This was achieved through a preliminary screening of free lutein degradation and is described in detail in Supporting Discussion 1.

### Chemical Stability of Model Lutein Diesters in the Absence of Chemical Interferences

**Figure 1:**
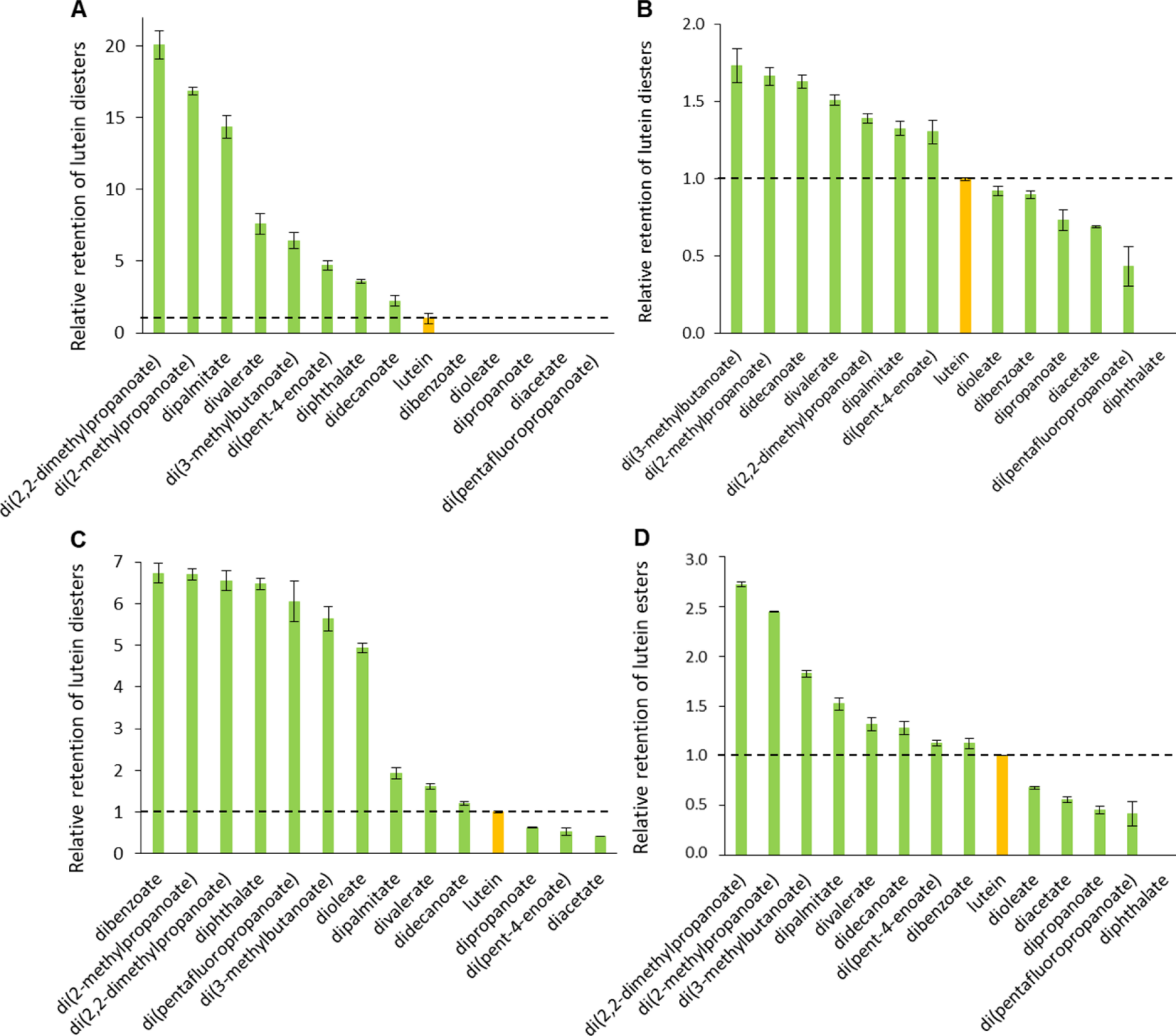
Stability of synthesized lutein diesters in comparison to free lutein. The presented data reflect the retention of compounds after 7 days of exposure to different stress conditions: elevated temperature—60 °C (A), light—366 nm (B), oxidant—H_2_O_2_, (C) and an acidic medium (D). Ethanolic solutions of pure lutein diesters were incubated in the absence of any interfering compounds (exact conditions are described in the Experimental Section). The dotted horizontal line highlights the retention of lutein at unity, and error bars depict the standard deviation of analytical measurements.

Thirteen distinct lutein diesters (lutein diacetate, dipropanoate, di(2,2-dimethylpropanoate), di(2-methylpropanoate), di(3-methylbutanoate), divalerate, di(pent-4-enoate), dibenzoate, didecanoate, dipalmitate, dioleate, di(pentafluoropropanoate), and lutein diphthalate) (Table 1) were synthesized and their stability evaluated in an ethanolic medium, in which both free xanthophylls and xanthophyll esters (as well as carotenes) are sufficiently soluble.^30^Figure 1 shows the retention of studied lutein diesters after their exposure to four individual stress conditions over a 7 day period. The data were normalized to free lutein to highlight the relative influence of a particular carboxylic acid (structural/electronic effect) on the stability of the resulting lutein diester. The results of the time course experiments are also presented in Figures S5–S12 to illustrate the degradation kinetics. The control samples for the four data sets (four stress conditions) demonstrated excellent analyte recovery (95–104%, *n* = 52), ensuring that the observed pigment degradation was primarily induced by the applied stress condition. In general, esterification altered the degradation kinetics of lutein, but the sign and magnitude of the change was not trivial in all cases.

**Table 1:**
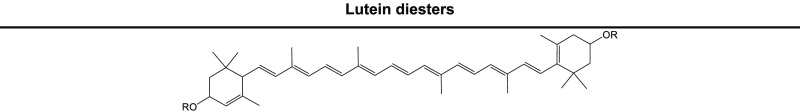

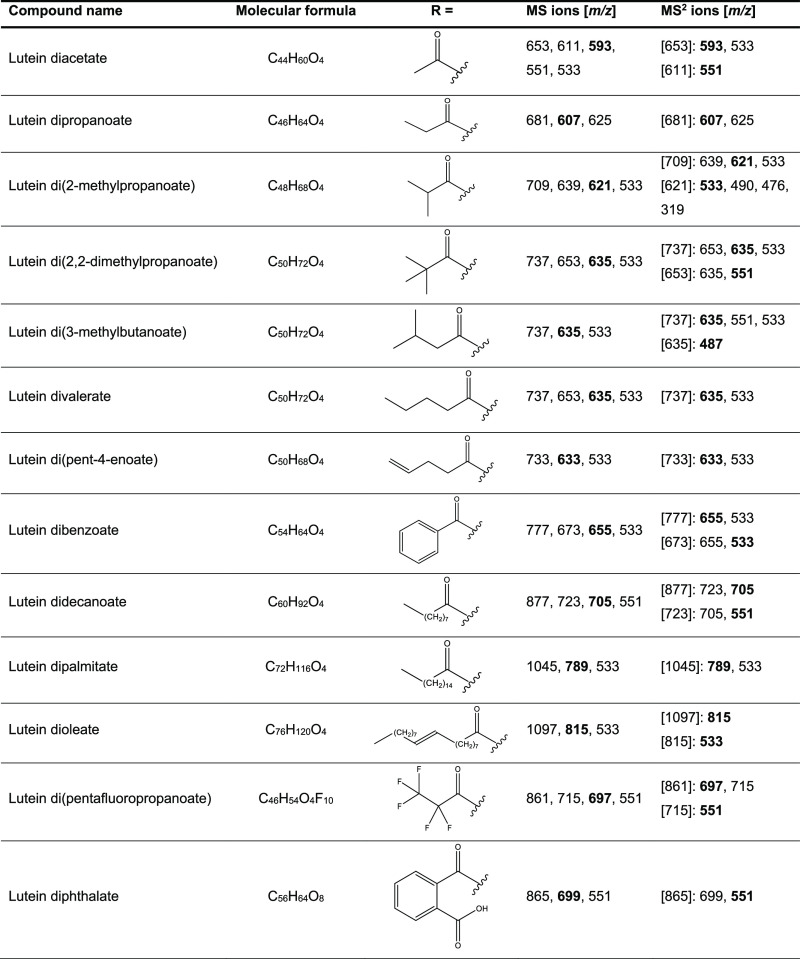
Lutein Diesters Used in the Forced Degradation Study with Accompanying MS and MS^2^ Data^a^

a Most abundant spectral signals are in boldface.

#### Temperature

Thermal stability is a property of lutein that was not greatly affected by esterification. At 60 °C, lutein diphthalate was the least stable lutein product, showing total degradation after merely 2 days of exposure. Lutein di(pentafluoropropanoate), diacetate, dipropanoate, dibenzoate, and dioleate showed higher resistance, but compared to free lutein, esterification still resulted in destabilization of the parent compound. For the latter two diesters, however, the difference was <10% (Figures 1A and S5). Other diesters demonstrated somewhat higher stability, with lutein di(3-methylbutanoate) giving the best results (a 70% improvement over free lutein). Although esterified xanthophylls are generally thought to have improved thermal resistance,^22,28,31,32^ the results presented above do not support such a generalization. It has been previously shown that the stability of astaxanthin increases with the degree of esterification (diester > monoester > free astaxanthin) and with the length of the alkyl chain.^24,25^ However, no such direct correlation was found here, as both short- and long-chain aliphatic lutein diesters demonstrated an incidental (de)stabilizing effect on the core xanthophyll (Figures 1A and S5). The degree of saturation of the carboxylic acid used, on the other hand, might be a parameter that should be further investigated, as lutein divalerate and lutein dipalmitate showed less degradation compared to lutein di(pent-4-enoate) and lutein dioleate, respectively. Similar conclusions were drawn for astaxanthin esterified with monounsaturated and polyunsaturated fatty acids.^25^ It should also be mentioned that interconversion of all-*trans* lutein diesters to *cis* derivatives was observed (Figure S6), supporting geometric isomerization as a major degradation pathway. This undesirable transformation of xanthophylls has been previously demonstrated for similar systems in temperature-induced degradation studies.^22,33^

#### Light

The photosensitivity of xanthophylls is a well-recognized concern in many areas of carotenoid research,^34^ and esterification has been suggested as a possible strategy to mitigate this problem.^22,35−38^ Esterification may perhaps also lead to alternative pigment decomposition mechanisms, as the more stable astaxanthin palmitate has been shown to convert mainly to the 13-*cis* isomer upon UV irradiation, whereas the 9-*cis* isomer was the major degradation product of the less-stable free astaxanthin.^23^ Interestingly, in a separate study, β-cryptoxanthin palmitate exhibited inferior photostability compared to the free xanthophyll,^26^ which immediately raises the question: does the core xanthophyll, and not just the selection of an appropriate carboxylic acid, also affect the behavior of a particular xanthophyll ester? Here, we present only the data for lutein diesters and show that lutein dibenzoate, dioleate, dipropanoate, diacetate, and di(pentafluoropropanoate) completely degraded under a UV-A light source (365 nm) after 7 days, while 2.1% of free lutein remained (Figures 1B and S7C,D). The other eight esterification attempts resulted in increased stability of lutein (from about 2-fold to 21-fold). The most significant improvement was observed for lutein di(2,2-dimethylpropanoate), lutein di(2-methylpropanoate), and lutein dipalmitate (in descending order) (Figures 1B and S7A). Diesters with unsaturated alkyl chains were again found to be less stable. The difference is particularly large when the lutein dipalmitate/lutein dioleate pair is considered (Figures 1B and S7B,C). Levels of the *cis*-lutein diesters either remained essentially constant during the 7 day experiment (lutein divalerate, di(2,2-dimethylpropanoate), didecanoate, di(3-methylbutanoate), and dipalmitate) or decreased in accordance with the degradation of the all-*trans* species, but in no case did the absolute value increase (Figure S8). To explain the stable concentration of *cis*-isomers, the rate of *trans* → *cis* isomerization must be commensurate with the rate of *cis*-isomer degradation, that is, cleavage of the conjugated double bond that forms the backbone of xanthophyll. When the amount of *cis*-lutein diesters decreased with time, the rate of their degradation must have been higher than the rate of their formation from the original all-*trans* forms.

#### Oxidants

Upon exposure to H_2_O_2_, lutein diacetate, di(pent-4-enoate), and dipropanoate degraded faster than free lutein (Figures 1C and S9D), whereas lutein dibenzoate, di(2-methylpropanoate), di(2,2-dimethylpropanoate), diphthalate, di(pentafluoropropanoate), di(3-methylbutanoate), and dioleate all showed a significant improvement in stability (5- to 7-fold) (Figures 1C and S9A,B). Similar to the light-induced degradation experiments, no geometric isomerization was observed; on the contrary, the low initial concentrations of the *cis*-species (side products of the synthesized all-*trans* lutein diesters) continued to decrease over time for most of the compounds studied (Figure S10). Most importantly, highly fluorinated or aromatic derivatives exhibited excellent resistance to oxidation by H_2_O_2_, whereas poor stability of these compounds (relative to free lutein) was generally observed under all other stress conditions studied here (Figure 1A,B,D). To the best of our knowledge, there are no reports of systematic forced degradation of xanthophyll esters by hydrogen peroxide, so no relevant comparison with data from the literature is possible.

#### Acidic Medium

Strong acids such as sulfuric and trifluoroacetic acids form ion pairs with carotenoids and eventually cause their degradation.^39,40^ The mechanism of degradation by weak acids is still debated, but one thing is certain: protonation of a (conjugated) double bond facilitates geometric isomerization (Figure S11).^39^ Therefore, the observed increase in the content of *cis*-derivatives of lutein diesters at low pH was not surprising (Figure S12). With minor deviations, the stability order for all 13 studied compounds closely resembled that obtained under thermal stress (Figure 1A,D), indicating similar degradation pathways. Carboxyl functionality was previously suggested to increase the stability of astaxanthin succinate at pH < 3,^25^ but the only acidic derivative in our study (lutein diphthalate) completely degraded after 7 days (Figures 1D and S11D). Lutein di(pentafluoropropanoate), dipropanoate, diacetate, and dioleate performed slightly better, although they still exhibited a destabilizing effect. Lutein di(2,2-dimethylpropanoate), di(2-methylpropanoate), and di(3-methylbutanoate) showed the highest stability with about 2- to 3-fold improvement relative to free lutein (Figures 1D and S11A). Lutein dipalmitate, divalerate, didecanoate, di(pent-4-enoate), and dibenzoate showed only modest change (up to 1.5-fold improvement) (Figures 1D and S11B,C). Interestingly, in a study by Hadjal et al. no significant differences in stability under acidic conditions (pH 3.5) were observed between free hydroxy xanthophylls (lutein, β-cryptoxanthin, and zeaxanthin) and their esterified forms found in blood oranges.^28^

The above results show that improving the chemical stability of lutein by esterification is highly intricate and the strategy should ideally be tailored to the intended environment (or use) of the lutein ester. Not many general structure–property relationships could be established and, more importantly, we show that not every esterification attempt results in successful protection of lutein. Lutein diacetate and lutein dipropanoate were highly unstable regardless of the stress condition used and should be avoided. Lutein dioleate, on the one hand, showed good resistance to hydrogen peroxide, but it degraded even faster than free lutein when exposed to light, elevated temperatures, or an acidic medium. The same trend was observed for fluorinated and aromatic diester derivatives, with lutein dibenzoate in particular showing the highest resistance to oxidation of all 13 model lutein diesters. The degradation rate of lutein diesters was roughly inversely proportional to the length of their linear saturated aliphatic chains, with some exceptions [e.g., lutein dipalmitate (Figure 1A), lutein didecanoate (Figure 1B,C), and lutein diacetate (Figure 1D)]. Finally, esterification with branched short-chain aliphatic carboxylic acids proved to be the best overall strategy for stabilizing lutein. Regardless of the stress conditions applied, lutein di(2,2-dimethylpropanoate), di(2-methylpropanoate), and di(3-methylbutanoate) showed 1.5- to 21-fold higher stability relative to free lutein, and at least two of them were always among the three most stable lutein diester products (Figure 1A–D). It should be stressed that in general the process of esterification most efficiently protected lutein from photodegradation and oxidation, while it was less effective in preventing light- and acid-induced degradation. The presented results in many cases diverge with published reports, as the final outcome of a forced degradation study depends on many variables, including the dissolution medium used and the influence of the sample matrix (vide infra).

### Chemical Stability of Model Lutein Diesters within the Japanese Knotweed Green Leaf Matrix

Most common xanthophylls are now commercially available in the pure crystalline form, but their high price prevents their broad use in industrial applications. For this reason, many xanthophyll-based food supplements or cosmetic products are based on natural plant extracts, but even these have some drawbacks. For example, cultivation of *Tagetes erecta* (marigold), the world’s most important natural source of lutein, is demanding and requires two to three times more energy and financial resources (fertilizers, pesticides, etc.) than cultivation of food crops such as maize (*Zea mays* L.).^41^ As the human population increases, this “flower cultivation” is being challenged in terms of socioeconomic sustainability, so new renewable sources of lutein are being actively sought.^42^ We have recently shown that lutein extracted from the leaves of Japanese knotweed can be effectively used to prepare lutein diesters.^6,19^ Because the plant is invasive and has a biomass yield per hectare that is three and four times higher than that of corn and wheat, respectively,^4,43^ Japanese knotweed represents a ubiquitous and virtually unlimited source of lutein. In the previous section, we showed that esterification alters the chemical stability of lutein, but two questions remain. Can the demonstrated properties of lutein diesters prepared from pure compounds be equated with those of a corresponding diester synthesized from the Japanese knotweed extract, and if not, what is the role of the plant matrix in this process?

To synthesize lutein diesters, free lutein was first released from Japanese knotweed leaf tissue using green supercritical CO_2_ (sc-CO_2_) extraction, which had previously been shown to be a sustainable and efficient approach for extracting carotenoids and other lipophilic compounds from many natural materials.^44−46^ By using sc-CO_2_, cleaner carotenoid extracts can be obtained with less potential chemical interferences compared to extracts obtained with other common solvents such as acetone, EtOAc, EtOH, and similar.^19^ For the synthesis of the 13 model lutein diesters in the next step, the sc-CO_2_ extract was used without further purification to avoid the generation of additional waste; from the perspective of economic and environmental sustainability, this is very important during scale-up and industrial implementation. After completion of the reaction, the lutein diester products were purified and excess reagents were quantitatively removed, but a moderate chemical background derived from the Japanese knotweed matrix remained. Some of the nucleophilic compounds from the matrix inevitably reacted with the various acid anhydrides used, producing unique compounds that contributed to the diversity of environments of the individual lutein diesters (Figure S13). The background of the 13 synthesized lutein diesters also varied slightly with the purification protocol used. Because it was not possible to identify all matrix components and account for their direct or indirect interfering effects on the stability data, no direct comparison was made between individual lutein diesters. Instead, the rate of degradation of a particular lutein diester was evaluated based on the origin of the lutein from which it was synthesized. In this way, the effect of the Japanese knotweed matrix could be selectively evaluated. For simplicity reasons, in the next section lutein diesters prepared from the commercially obtained lutein will be referred to as “pure lutein diesters”, and those prepared from the Japanese knotweed green leaf extract will be referred to as “extract lutein diesters”.

#### Temperature

**Figure 2:**
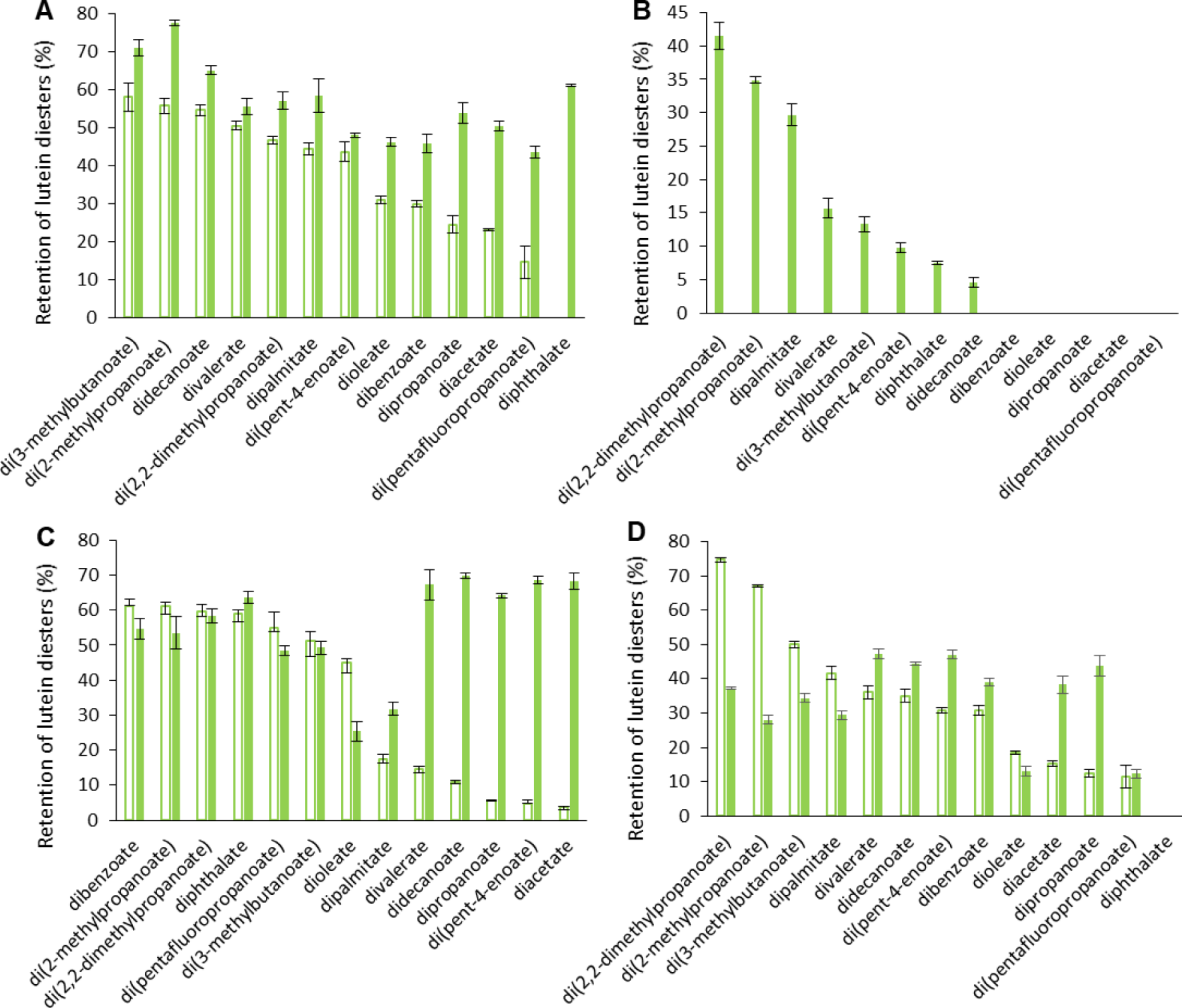
Stability of synthesized lutein diesters in the absence (empty columns) and presence (filled columns) of interfering compounds from the sc-CO_2_ extract of Japanese knotweed green leaves. The presented data reflect the retention of compounds after 7 days of exposure to different stress conditions: elevated temperature—60 °C (A), light—366 nm (B), oxidant—H_2_O_2_ (C), and an acidic medium (D). Error bars depict the standard deviation of analytical measurements.

All 13 lutein diesters synthesized from the Japanese knotweed leaf extract showed a consistently better stability at 60 °C (20% higher on average) compared with the same compounds prepared from pure lutein (Figures 2A, S14 and S15). This improvement was most evident for the least stable pure lutein diesters, especially for lutein diphthalate, where 61% compound retention was achieved after 7 days in contrast to the complete degradation observed for the pure lutein diester. A significant increase in thermal resistance of more than 2-fold was also evidenced for lutein di(pentafluoropropanoate), diacetate, and dipropanoate. The most stable extract lutein diester proved to be lutein di(2-methylpropanoate) with pigment retention of more than 77%.

#### Light

The matrix of the Japanese knotweed extract had an immense negative impact on the photostability of lutein diesters as nearly all completely degraded after only 2 days of UV irradiation, with only lutein diphthalate showing a minimal retention of 8% (Figures 2B, S16 and S17).

#### Oxidants

Upon exposure to H_2_O_2_, not all extract lutein diesters followed the same shift in stability both in terms of the direction and extent of the change (Figures 2C, S18 and S19). Lutein dibenzoate, di(2-methylpropanoate), di(2,2-dimethylpropanoate), di(pentafluoropropanoate), and di(3-methylbutanoate) were slightly less stable compared to their pure analogues, but this difference was within experimental error in most cases. Lutein dioleate was the only product whose stability deteriorated by up to 45% after 7 days. The rest of the extract lutein diesters showed a higher resistance relative to pure lutein diesters, particularly lutein dipropanoate, di(pent-4-enoate), diacetate, didecanoate, and divalerate, which degraded 4.5 to 17 times more slowly.

#### Acidic Medium

When we introduce lutein diesters into the chemical environment of Japanese knotweed and expose them to an acidic medium, we again get heterogeneous results (Figures 2D, S20 and S21). Lutein diphthalate completely degraded after 7 days, but then, the same result was obtained for the corresponding pure lutein diester. For most other lutein diesters, the relative changes in stability were not substantial (8–32%). The four exceptions were lutein di(2,2-dimethylpropanoate) and di(2-methylpropanoate), for which 51 and 58% higher degradation was observed, respectively, and lutein diacetate and dipropanoate, for which a 153 and 266% increase in stability, respectively, was determined.

In summary, the use of the green leaf extract of Japanese knotweed as a starting material for the preparation of lutein diesters certainly introduces another variable that should not be ignored. The stability of a synthesized compound is either increased or decreased if not all matrix components are completely removed (Figure 2). For instance, the plant matrix strongly promoted photodegradation of all lutein diesters while further protecting them at elevated temperatures. When exposed to oxidants and acids, the effect of the matrix on aromatic and unsaturated aliphatic lutein diesters was random. On the other hand, a stable increase in pigment retention was observed for linear aliphatic derivatives, whereas branched short-chain diesters showed a consistent reduction in stability. If generalization is allowed, the leaf matrix of Japanese knotweed appears to balance the retention of the compounds studied, increasing the stability of the most labile pure lutein diesters and causing more rapid degradation of the most stable ones. Direct comparisons between the individual extract lutein diesters can only be made tentatively due to the nuances in their respective environments as explained above. Matrix matching was intentionally omitted because it does not support a potential implementation of the technology in practice. However, if necessary, the Japanese knotweed extract could be subjected to additional downstream processing before or after the esterification reaction. In this way, the stability data for the pure lutein diesters presented in Figure 1 could be reproduced, but on account of burdening the environment by generating additional waste. A lower stability of extract lutein diesters evidenced in this study could arguably be linked to the presence of endogenous acids of Japanese knotweed (palmitic, myristic, stearic, oleic, and lauric acid).^19^ In contrast, the observed improvements in stability most likely reflect the presence of other antioxidants such as chlorophylls, other xanthophylls, and polyphenols, although the latter compounds could not be detected in the plant extract due to the hydrophobic nature of the extraction fluid (sc-CO_2_). Nevertheless, using the antioxidant 2,2-diphenyl-1-picrylhydrazyl (DPPH) assay, we confirmed that at the same lutein concentration levels, the sc-CO_2_ extract had higher antioxidant activity (IC_50_ = 660 μg/L) compared to free lutein in solution (Figure S22). The same trend was also confirmed for one model lutein diester—lutein diacetate. Finally, stability data for free lutein (both pure and from plant extract) were intentionally omitted from Figure 2 because they would provoke an unwarranted, yet instinctive, comparison with lutein diesters. Nonetheless, it should be mentioned that unesterified lutein from Japanese knotweed generally showed 25% higher stability on average compared with pure free lutein, but as with lutein diesters, free lutein within the plant extract matrix degraded very rapidly when exposed to light (Figure S23).

It has been shown before that the relative stability of xanthophylls can be highly dependent on the matrix.^28^ Good examples of this are lutein and β-cryptoxanthin, as their relative degradation rates have been shown to change order when tested in two different systems—citrus juice and virgin olive oil.^47,48^ Thus, the relative stability data of pure model xanthophylls, either free or esterified, can rarely be used to predict their stability in any given food or plant matrix.^28^ The kinetic data of our 13 model lutein diesters are in complete agreement with this observation because many of them showed different behavior when synthesized and assessed within the Japanese knotweed matrix.

### Chemical Stability of Free Capsanthin, Zeaxanthin, Violaxanthin, and β-Cryptoxanthin

The above results illustrate the intricate relationship between the chemical stability of a particular lutein diester, its molecular structure, and its environment. To obtain a first indication of whether the observed rules can be extended to other known xanthophylls, four additional free pigments were subjected to forced degradation: capsanthin, violaxanthin (an epoxycarotenoid), zeaxanthin (lutein structural isomer), and β-cryptoxanthin (a monohydroxyxanthophyll). The results are shown in (Figures S24–S26). All four xanthophylls exhibited similar thermal degradation profiles and kinetics, with 31–53% xanthophylls remaining after 7 days (Figures S24A and S25A). Upon irradiation with UV light, lutein could be clearly distinguished from the other compounds as it degraded much faster (Figures S24B and S25B). Its structural isomer zeaxanthin, which contains an extra conjugated double bond, displayed about 80-fold better resistance. All xanthophylls were unstable under oxidative stress (<10% remaining after 7 days), but the monohydroxyxanthophyll β-cryptoxanthin completely degraded after only 4 days of exposure (Figures S24C and S25C). Finally, violaxanthin could be singled out during forced degradation in acidic media, as no compound could be detected in the solution after only 24 h (Figures S24D and S25D). Epoxyxanthophylls have previously been shown to undergo ring-opening reactions under acidic conditions, causing degradation and a decrease in absorption in the 450 nm range.^49,50^ Although the studied xanthophylls possess the same (or a very similar) conjugated backbone, the above outliers indicate distinct degradation mechanisms in certain cases. This means that direct extrapolation of data on the potential stability of one xanthophyll to another should be done with caution, as esterification of individual pigments may not lead to the same stabilizing effect, especially when pigments are integrated into different real-life matrices.

## Conclusions

Lutein diesters are considered potential high-value products with superior chemical stability and a longer shelf-life compared to free lutein. Moreover, the lutein-rich leaves of Japanese knotweed may be a key ingredient in the production of such lutein diesters, as valorization of these plant wastes supports long-term economic and environmental sustainability. Nevertheless, such implementation is not without challenges. Here, we provide some missing evidence that adds to the current knowledge of structure–property relationships of xanthophyll esters. First, we have demonstrated that not all lutein esterification attempts lead to an improvement in its chemical stability; however, branched, short-chain derivatives were clearly the most promising. To complicate matters further, the success of a particular stabilization attempt was also dependent on the stress condition to which a particular lutein diester was exposed (heat, light, oxidant, or acid). Most importantly, we show that when using leaf extracts of Japanese knotweed to produce lutein diesters, the role of the plant matrix should not be ignored because it alters the degradation kinetics quite drastically in some cases. Although these effects may appear to be random, we observed that the plant matrix generally decreased the stability of most resilient pure lutein diesters, while it considerably increased the stability of those that essentially completely degraded in the absence of any interfering species. Therefore, the leaf matrix may not necessarily have a negative connotation, and its effect should be evaluated individually for each combination of lutein diester and stress condition. For these compounds to be fully recognized in the food supplement industry, further efforts should be made to investigate other esterification-induced changes in lutein, such as antioxidant activity, potential toxicity, and bioaccessibility, because chemical stability is only one, albeit important, aspect to be considered in the development of new bioactive compounds. For now, one thing is certain: the production of chemically stable lutein diesters from the waste material of Japanese knotweed is certainly feasible, but only the right strategy can lead to a favorable outcome.

## Supporting Information Available

Experimental details including materials, determination of antioxidant activity, and description of chromatographic methods used; degradation data on lutein and lutein diesters; Japanese knotweed background; and graphical presentation of the DPPH scavenging effect (PDF)
