# Correction to “Esterification of Lutein from Japanese Knotweed Waste Gives a Range of Lutein Diester Products with Unique Chemical Stability”

**DOI:** 10.1021/acssuschemeng.3c06864

**Published:** 2023-11-08

**Authors:** Valentina Metličar, Alen Albreht

Below, in the corrected Figure 1 of our original article, graphs A and B have been swapped to match the data and information presented in the figure caption, the main article text, and the Supporting Information (specifically, Figures S5–S12). Related to the Figure 1 correction, the Figure S5 caption has been revised so that lutein di(pentafluoropropanoate) is depicted by a “triangle” in Figure S5D and lutein diphthalate by a “square”.

**Figure 1:**
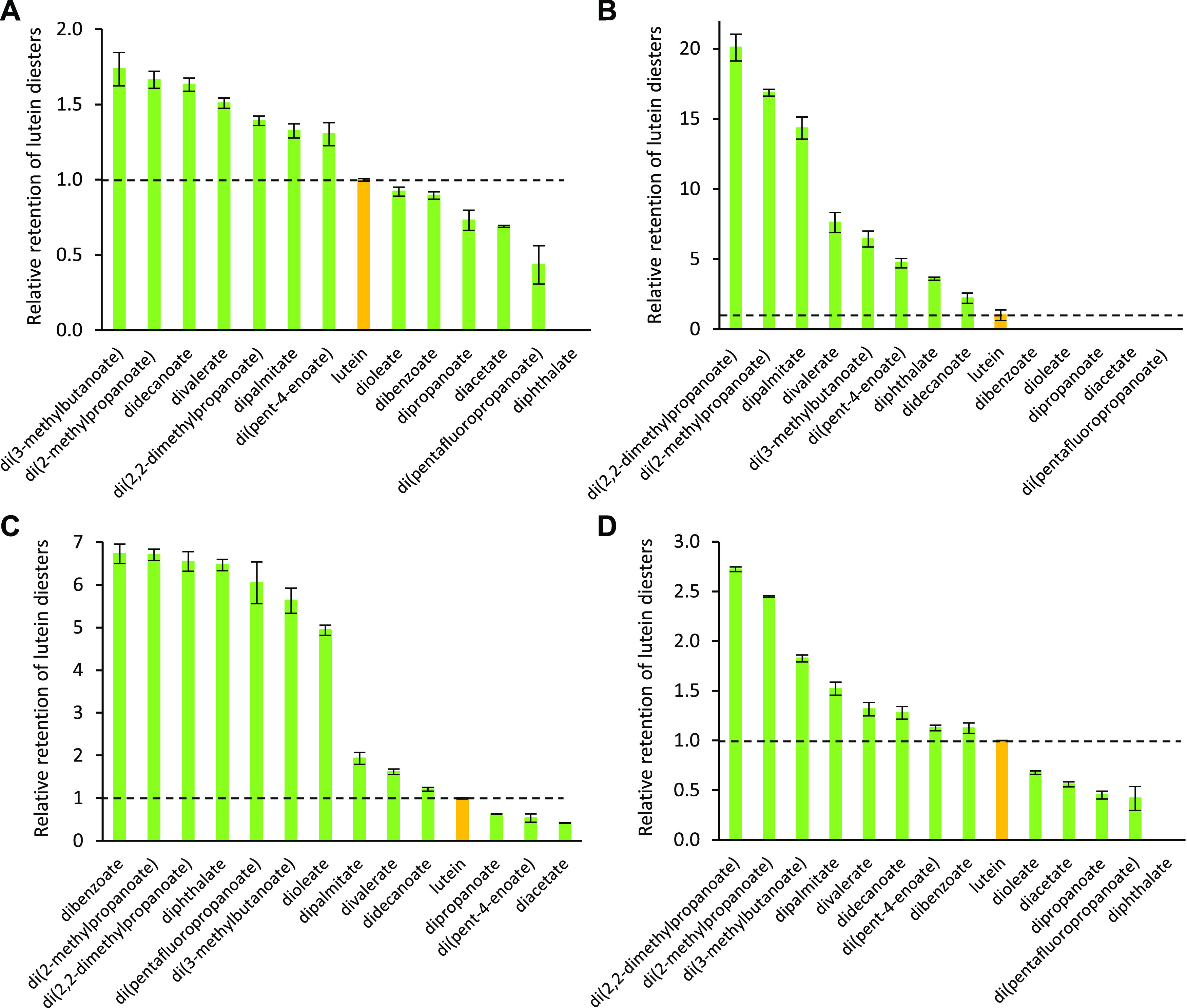
Stability of synthesized lutein diesters in comparison to free lutein. The presented data reflect the retention of compounds after 7 days of exposure to different stress conditions: elevated temperature—60 °C (A), light—366 nm (B), oxidant—H_2_O_2_ (C), and an acidic medium (D). Ethanolic solutions of pure lutein diesters were incubated in the absence of any interfering compounds (exact conditions are described in the Experimental Section). The dotted horizontal line highlights the retention of lutein at unity, and error bars depict the standard deviation of analytical measurements.

Below, in the corrected Figure 2 of our original article, the graph columns of panel B have been made hollow as they represent the data in the absence of interfering compounds, not in the presence of the latter. All other data in the figures are correct as originally published. No other content or conclusion of the published paper is affected.

**Figure 2:**
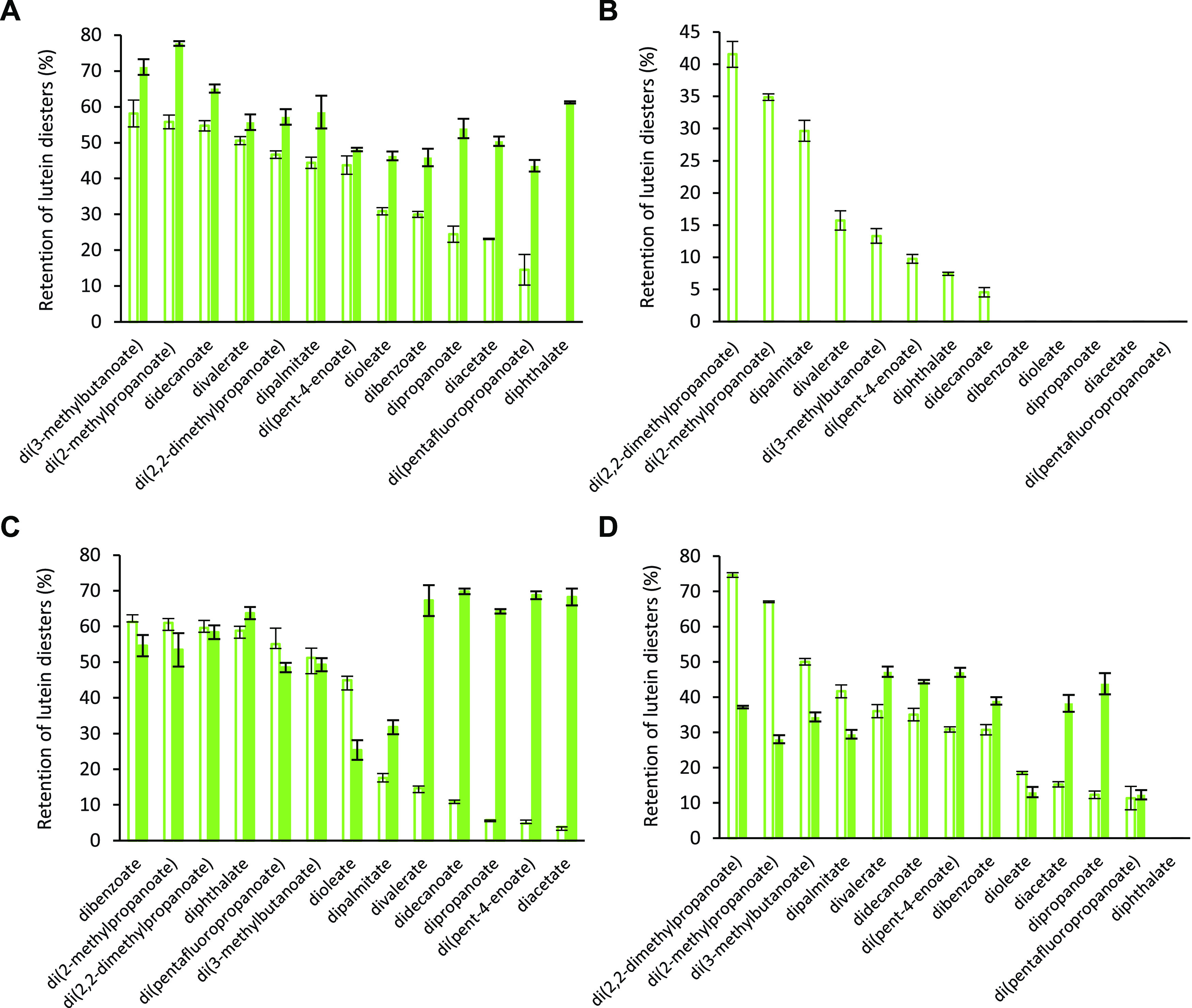
Stability of synthesized lutein diesters in the absence (empty columns) and presence (filled columns) of interfering compounds from the sc-CO_2_ extract of Japanese knotweed green leaves. The presented data reflect the retention of compounds after 7 days of exposure to different stress conditions: elevated temperature—60 °C (A), light—366 nm (B), oxidant—H_2_O_2_ (C), and an acidic medium (D). Error bars depict the standard deviation of analytical measurements.

## Supporting Information Available

Same description as in the original article (PDF)

