# Supplementary material for: Correction to “Esterification of Lutein from Japanese Knotweed Waste Gives a Range of Lutein Diester Products with Unique Chemical Stability”
Source: ACS Sustain Chem Eng. 2023 Nov 8;11(46):16713–5. doi: 10.1021/acssuschemeng.3c06864 (PMC10664140; doi:10.1021/acssuschemeng.3c06864)
Supplement: Supplementary file 1 — sc3c06864_si_001.pdf [file sc3c06864_si_001.pdf]

Esterification of Lutein from Japanese Knotweed Waste Gives a Range of Lutein Diester Products with Unique Chemical Stability

Valentina Metličar[a,b] and Alen Albreht*[a]

[a] Laboratory for Food Chemistry, Department of Analytical Chemistry, National Institute of Chemistry, Hajdrihova 19, SI-1000 Ljubljana, Slovenia

[b] Faculty of Chemistry and Chemical Technology, University of Ljubljana, Večna pot 113, SI-1000 Ljubljana, Slovenia

* To whom correspondence should be addressed: Alen Albreht, Laboratory for Food Chemistry, Department of Analytical Chemistry, National Institute of Chemistry, Hajdrihova 19, SI-1000 Ljubljana, Slovenia; alen.albreht@ki.si; Tel: 038614760269

Number of pages: 35

Number of Figures: 26

Number of Schemes: 1

TABLE OF CONTENTS

Experimental……………………………………………………………………………………..S3
Supporting discussion ……………………………………………………………………………S7
Supporting Shemes…………………………………………………………………………………S8
Sheme S1………………………………………………………………………………………………S8
Supporting Figures…………………………………………………………………………………S9
Figure S1………………………………………………………………………………………………S9
Figure S2 ……………………………………………………………………………………………S10
Figure S3………………………………………………………………………………………………S11
Figure S4………………………………………………………………………………………………S12
Figure S5………………………………………………………………………………………………S13
Figure S6………………………………………………………………………………………………S14
Figure S7………………………………………………………………………………………………S15
Figure S8………………………………………………………………………………………………S16
Figure S9………………………………………………………………………………………………S17
Figure S10……………………………………………………………………………………………S18
Figure S11……………………………………………………………………………………………S19
Figure S12……………………………………………………………………………………………S20
Figure S13……………………………………………………………………………………………S21
Figure S14……………………………………………………………………………………………S22
Figure S15……………………………………………………………………………………………S23
Figure S16……………………………………………………………………………………………S24
Figure S17……………………………………………………………………………………………S25
Figure S18……………………………………………………………………………………………S26
Figure S19……………………………………………………………………………………………S27
Figure S20……………………………………………………………………………………………S28
Figure S21……………………………………………………………………………………………S29
Figure S22……………………………………………………………………………………………S30
Figure S23……………………………………………………………………………………………S31
Figure S24……………………………………………………………………………………………S32
Figure S25……………………………………………………………………………………………S33
Figure S26……………………………………………………………………………………………S34
References…………………………………………………………………………………………S35

Experimental

Chemicals and Materials. All chemicals were at least of analytical grade. Ethanol (EtOH) was purchased from Carlo Erba (Val-de-Reuil, France). Acetone, methanol (MeOH, LC-MS grade) and acetonitrile (ACN) were supplied by Honeywell (Seelze, Germany). Ethyl acetate (EtOAc), methyl *tert*-butyl ether (MTBE), isopropanol, orthophosphoric acid (85%), ammonium acetate (≥98%) and aqueous ammonia solution (32%) were supplied from Merck (Darmstadt, Germany). 4-Dimethylaminopyridine (DMAP), ammonium formate (≥99%), formic acid (≥98%) hydrogen peroxide (H_2O_2, 30%, *v/v*), 2,2-diphenyl-1-picrylhydrazyl (DPPH), all anhydrides: acetic (99%), propionic (≥99%), isobutyric (2-methylpropionic, 97%), isovaleric (3-methylbutyric, ≥95%), valeric (97%), pent-4-enoic (98%), 2,2-dimethylpropionic (trimethylacetic, 99%), pentafluoropropionic (≥99%), phthalic (≥99%), benzoic (≥95%), decanoic (96%), oleic (95%), and palmitic anhydride (97%); and two xanthophylls: all-*trans*-lutein (95%) and all-*trans*-violaxanthin (≥90%) were acquired from Sigma-Aldrich (St. Louis, MO, USA). Other xanthophylls: all-*trans*-zeaxanthin (≥98%), all-*trans*-capsanthin (≥95%), and all-*trans*-β-cryptoxanthin (≥97%) were purchased from Extrasynthèse (Genay, France). Unless said otherwise, xanthophylls in this study assumed an all-(*trans*)-configuration. Ultrapure Milli-Q water (18 MΩ cm^{-1}) was used. All solvents, plant material extract, and standard and reagent solutions in this study were purged with argon before use.

Xanthophyll standard solutions (10 – 15 mg/L) were prepared by accurately weighing a corresponding amount of an individual xanthophyll (zeaxanthin, lutein, capsanthin, violaxanthin, or β-cryptoxanthin) and dissolving it in EtOH. The exact concentrations were determined spectrophotometrically by using the Beer-Lambert correlation along with the absorption coefficients of xanthophylls.(1) Before further use, the prepared solutions were stored in amber glass vials at –80 °C.

Antioxidant activity

Stock ethanolic solutions of lutein from sc-CO_2 extract of Japanese knotweed green leaves, lutein standard, and two lutein diacetate solutions (one synthesized from the pure lutein standard and the other from the sc-CO_2 extract of Japanese knotweed green

leaves) were diluted in series with EtOH to obtain 12 working solutions in the concentration range of 0.006 – 16 mg/L for lutein and lutein diacetate, respectively. Two milliliters of each individual working solution were transferred into amber 8 mL glass storage vial in triplicates to which 667 µL of 200 µM methanolic solution of DPPH was added. Prepared solutions were vortexed for 10 s and stored in amber storage vials in the dark (22 °C) for 30 min. Afterwards, spectrophotometric measurements at 517 nm (A_A) were performed by using a Lambda 45 UV/Vis spectrophotometer (Perkin Elmer, Waltham, MA, USA). To correct for the absorbance stemming from the sample itself (A_B), 667 µL of methanol (instead of DPPH) were added to the working solution. The DPPH controls (A_C) were prepared by adding the same amount of DPPH to ethanol (instead of a sample) in triplicate, while control for the double beam spectrophotometer (baseline correction) was prepared by adding MeOH into the sample solvent in the same ratio (667 µL into 2 mL). The graphs and IC50 values were obtained using GraphPad Prism 7 (2). DPPH scavenging effect was calculated based on the following equation:

DPPH scavenging effect (%) = $100 - ((A_A - A_B) \times 100/A_C)$; where:

A_A – absorbance of a working solution (after 30 min of DPPH addition)
A_B – absorbance of sample blank solutions (in the absence of DPPH)
A_C – absorbance of DPPH controls (sample omitted)

HPLC–PDA–MS^2 Analysis of Free Xanthophylls and Synthesized Lutein Diesters.
For high performance liquid chromatographic (HPLC) analyses, an Ultimate 3000 HPLC system (Thermo Fisher Scientific, Waltham, USA) coupled to an LCQ Fleet MS system (Thermo Fischer Scientific) with atmospheric-pressure chemical ionization (APCI) ion source in positive ion mode was used. The HPLC system consisted of a thermostated autosampler with a 100 µL syringe, a quaternary pump and a photodiode array (PDA) detector. Xcalibur software (version 4.0.27.42) was used for data evaluation of chromatograms. The compounds were separated on an Accucore™ C30 column (150 mm × 2.1 mm i.d., 2.6 µm) from Thermo Fischer (Waltham, USA) which was connected to a security guard column (C18 4 mm × 3 mm i.d.) from Phenomenex (Torrance, CA, USA). The mobile phase consisted of 95% MeOH (solvent A) and MTBE (solvent B) and

the following gradient program was used: 0 – 5 min (100% A), 5 – 15 min (100 – 45% A), 15 – 17 min (45% A), 17 – 18 min (45 – 25% A), 18 – 20 min (25% A), 20 – 22 min (25 – 100% A), followed by a 3 min re-equilibration step (100% A). The flow rate was maintained at 0.4 mL/min, while column and autosampler temperatures were set at 25 °C and 10 °C, respectively. Injection volume was 10 µL. Acquisition wavelengths were set to 210, 280, 340 and 450 nm. UV-VIS spectra were acquired in the range from 200–800 nm. APCI ion source conditions were as follows: transfer capillary temperature 300 °C, vaporizer temperature 300 °C, sheath gas flow rate 25 a.u., auxiliary gas flow rate 5 a.u., spray voltage 3 kV and discharge current 4 µA. MS and MS^2 spectra were acquired in 200 – 1500 *m/z* range and collision energy of 35% was used to fragment the target precursor ions during MS^2 experiments. Due to negligible perturbations of the xanthophyll chromophore, the same molar absorption coefficient was assumed for the free lutein and their corresponding diesters in quantitative analysis.

Assessing the Complexity of Japanese Knotweed Matrix Background by HPLC–PDA–CAD. Analyses were performed on a Vanquish UHPLC system (Thermo Scientific) with thermostated autosampler, binary pump, diode array detector (DAD) with a 60 mm light tube, and charged aerosol detector (CAD). The separation was done on an Accucore^{TM} C30 column (150 mm × 2.1 mm i.d., 2.6 µm) from Thermo Fischer connected to the Phenomenex security guard column (C18 4 mm × 3 mm i.d.). The chromatographic conditions were the same as the ones used for the analysis of free xanthophylls and synthesized lutein diesters, with some adaptations. Acquisition wavelengths were set to 210, 280 and 450 nm. UV-VIS spectra were acquired in the range from 200–600 nm. Evaporator temperature on CAD was set to 45 °C and data was collected at a frequency of 10 Hz. Chromeleon software version 7.2 was employed for data processing.

HPLC-PDA Analysis of Unreacted Acid Anhydrides, Their Corresponding Carboxylic Acids and DMAP. HPLC analysis of different acid anhydrides, their corresponding carboxylic acids and DMAP was carried out by using an HPLC-PDA Surveyor Plus system from Thermo Finnigan, comprising of a thermostated autosampler, a quaternary pump and a PDA detector with 5 cm LightPipe flow cell. A Phenomenex Synergi Hydro – RP C18 (80A) column (150 mm × 4.6 mm i.d., 4 µm) with a security

guard column (C18 4 mm × 3 mm i.d.) from Phenomenex was used. The mobile phase consisted of 0.1% aqueous orthophosphoric acid (Solvent A), ACN (Solvent B) and MTBE : ACN = 2 : 8 (solvent C). The applied elution gradient was: 0 – 3 min (100% A), 3 – 10 min (to 5% A 95% B), hold for 6 min, 16 – 18 min (to 100% C), hold for 8 min, 26 – 27 min (to 100% B), hold for 4 min, 31 – 32 min (back to 100% A), hold for 5 min. Column oven and autosampler temperature were set to 30 °C and 15 °C, respectively. Flow rate was 1.5 mL/min, injection volume was 10 μL. Acquisition wavelengths were set to 210 nm, 280 nm and 450 nm. UV-VIS spectra (190–650 nm) were also acquired. For data evaluation ChromQuest 5.0 software was used.

Supporting Discussion 1

Selection of Suitable Experimental Conditions for the Forced Degradation Study. In order to reliably assess the (de)stabilizing effect of a particular lutein esterification strategy, we first set out to define a robust and suitable experimental framework. This was achieved through a preliminary screening of free lutein degradation that resulted in compound retention of approximately 2 – 33% after 7 days in an ethanolic solution. Temperature of 60 °C was chosen as an optimum parameter value corresponding to a 33% lutein retention (Figure S1). As expected, the degradation rate increased with the temperature,(3–5) but at 80 °C the isomerization of *trans* lutein into its *cis* isomers was also more pronounced. At 40 °C, 62% of lutein remained after 7 days. To study the effect of light, daylight exposure and the use of two artificial light sources (UV-A (365 nm) and white light) were examined. A smaller proportion (18 – 39%) of the total lutein degraded under daylight conditions and white light (Figure S2), while under UV light (365 nm) almost complete degradation was observed after 7 days (> 98%). Hydrogen peroxide (0.015, 0.15, and 0.45%) was used as an oxidant for the forced oxidative degradation of lutein. The optimal retention of lutein (10%) after 7 days was obtained in the presence of 0.15% $H_2O_{2(aq)}$, while the degradation was too rapid and too mild at 0.45% and 0.015% levels of $H_2O_{2(aq)}$, respectively (Figure S3). Finally, stability of lutein was also studied in 95% ethanolic solutions containing 10 mM buffer in the pH range from 1.2 – 9 (Figure S4). Lutein was stable at high to medium pH, while it degraded more rapidly in an acidic environment. The optimum stress conditions were achieved when buffer at pH 2 was used for the preparation of the xanthophyll solution (73% degradation). The relative standard deviations of 0.2 – 1.1% and 0.2 – 1.8%, determined for the intraday and interday precision, respectively, demonstrated the robustness and suitability of the above methodologies for the forced degradation study of lutein diesters.

Supporting Schemes

Scheme S1. The outline of the forced degradation study; preparation of lutein diesters (A) and evaluation of xanthophyll stability (B). See Experimental for details.

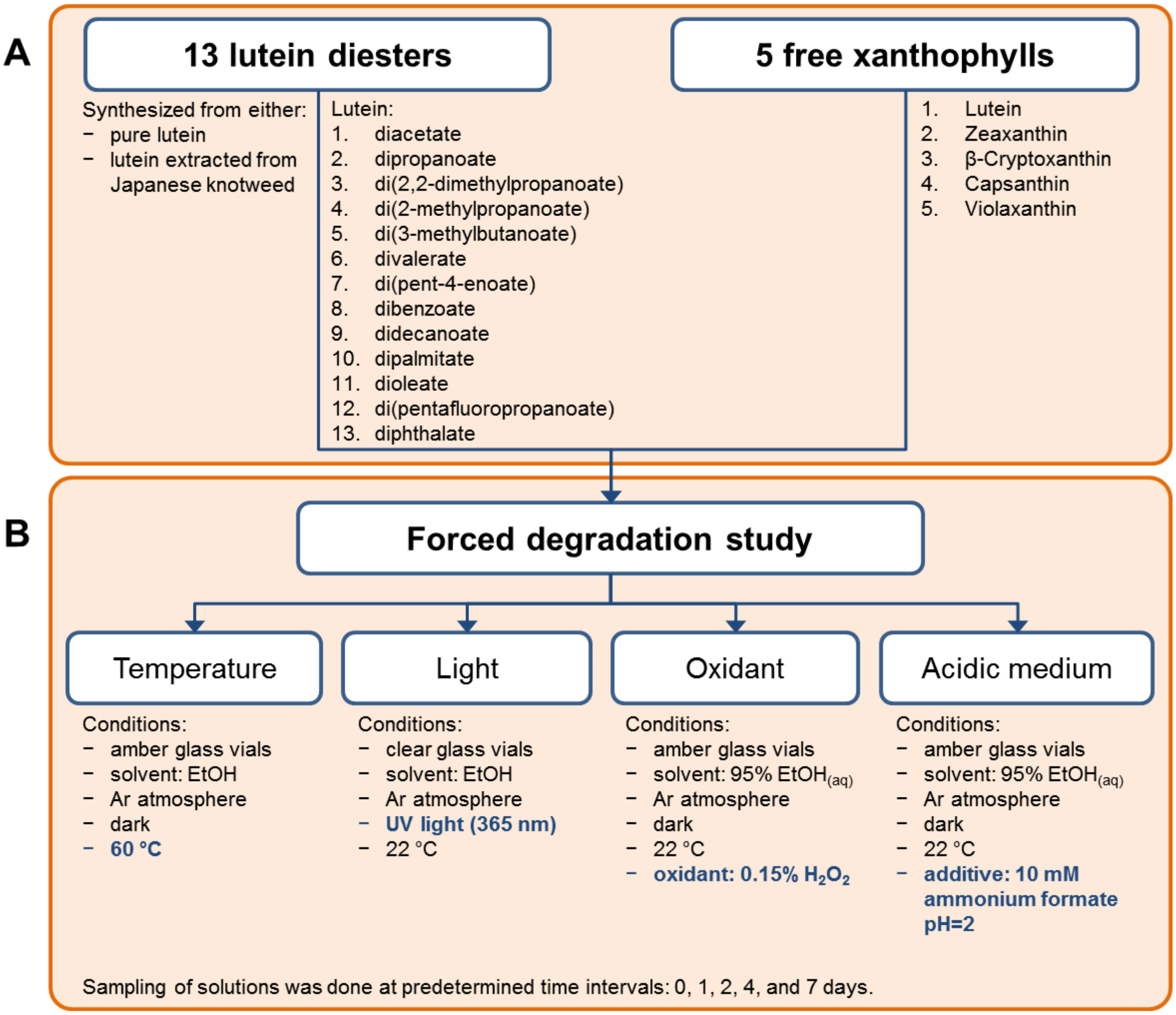

Supporting Figures

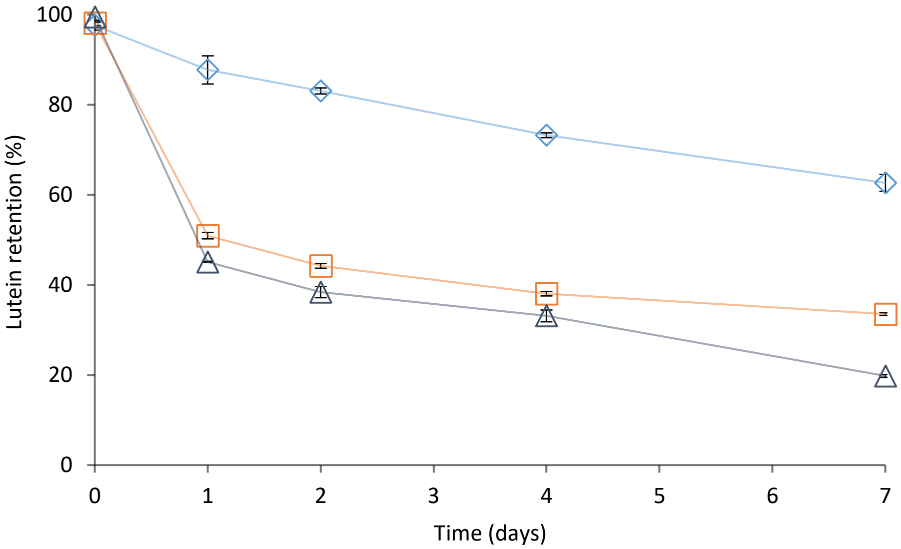

Figure S1. Lutein retention as a function of time at different temperatures: 40 °C (diamond), 60 °C (square) and 80 °C (triangle). A solution of lutein in ethanol (20 µM) was exposed to selected temperatures in an amber HPLC vial and under an argon atmosphere in the dark for 7 days. Experiments were carried out in triplicate.

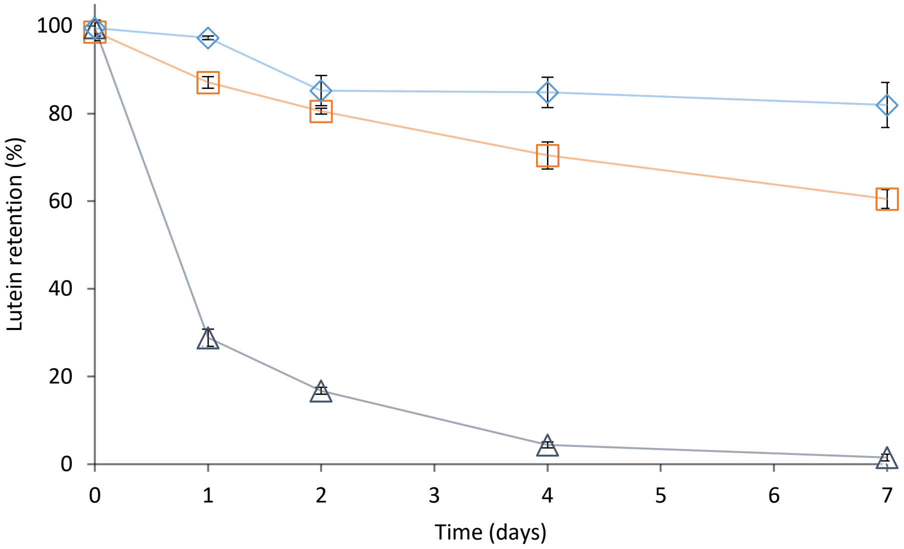

Figure S2. Lutein retention as a function of time at different illuminating conditions: daylight (diamond), visible light lamp (square) and 365 nm UV-A light (triangle). A solution of lutein in ethanol (20 μM) was exposed to selected illuminating conditions in a clear glass HPLC vial and under an argon atmosphere at 22 °C for 7 days. Experiments were carried out in triplicate.

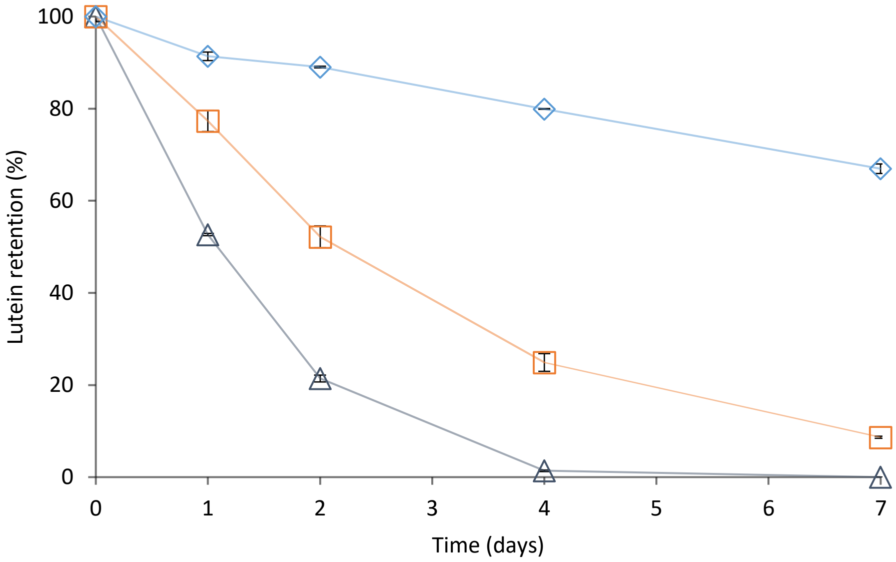

Figure S3. Lutein retention as a function of time at different concentrations of $H_2O_{2(aq)}$: 0.015% (diamond), 0.15% (square) and 0.45% (triangle). A solution of lutein in 95% $ethanol_{(aq)}$ (20 µM) was exposed to H_2O_2 in an amber HPLC vial at 22 °C and under an argon atmosphere in the dark for 7 days. Experiments were carried out in triplicate.

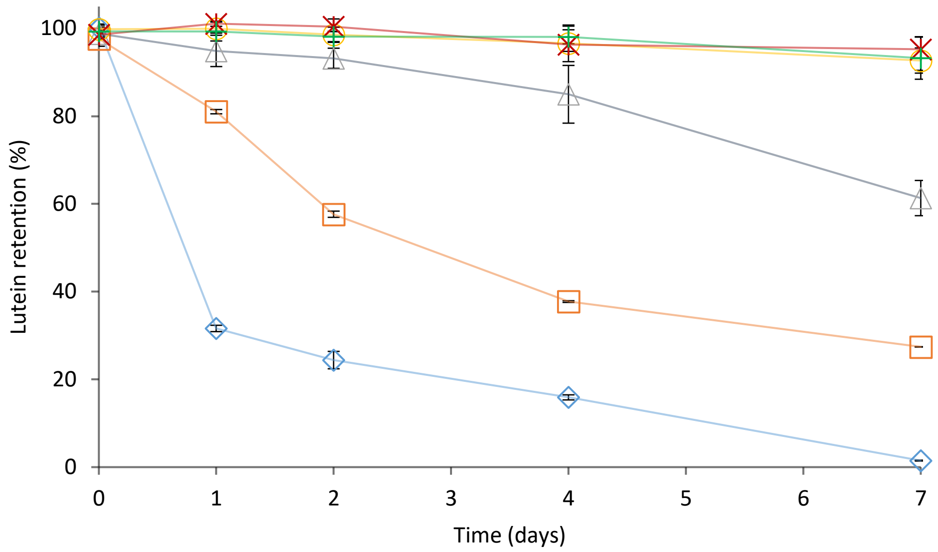

Figure S4. Lutein retention as a function of time at different pH conditions. A solution of lutein in 95% ethanol$_{(aq)}$ (20 µM), containing 1% formic acid (diamond) or 10 mM buffer at pH = 2 (square), pH = 3 (triangle), pH = 5 (circle), pH = 7 (asterisk), and pH = 9 (cross), was incubated in an amber HPLC vial at 22 °C and under an argon atmosphere in the dark for 7 days. Experiments were carried out in triplicate.

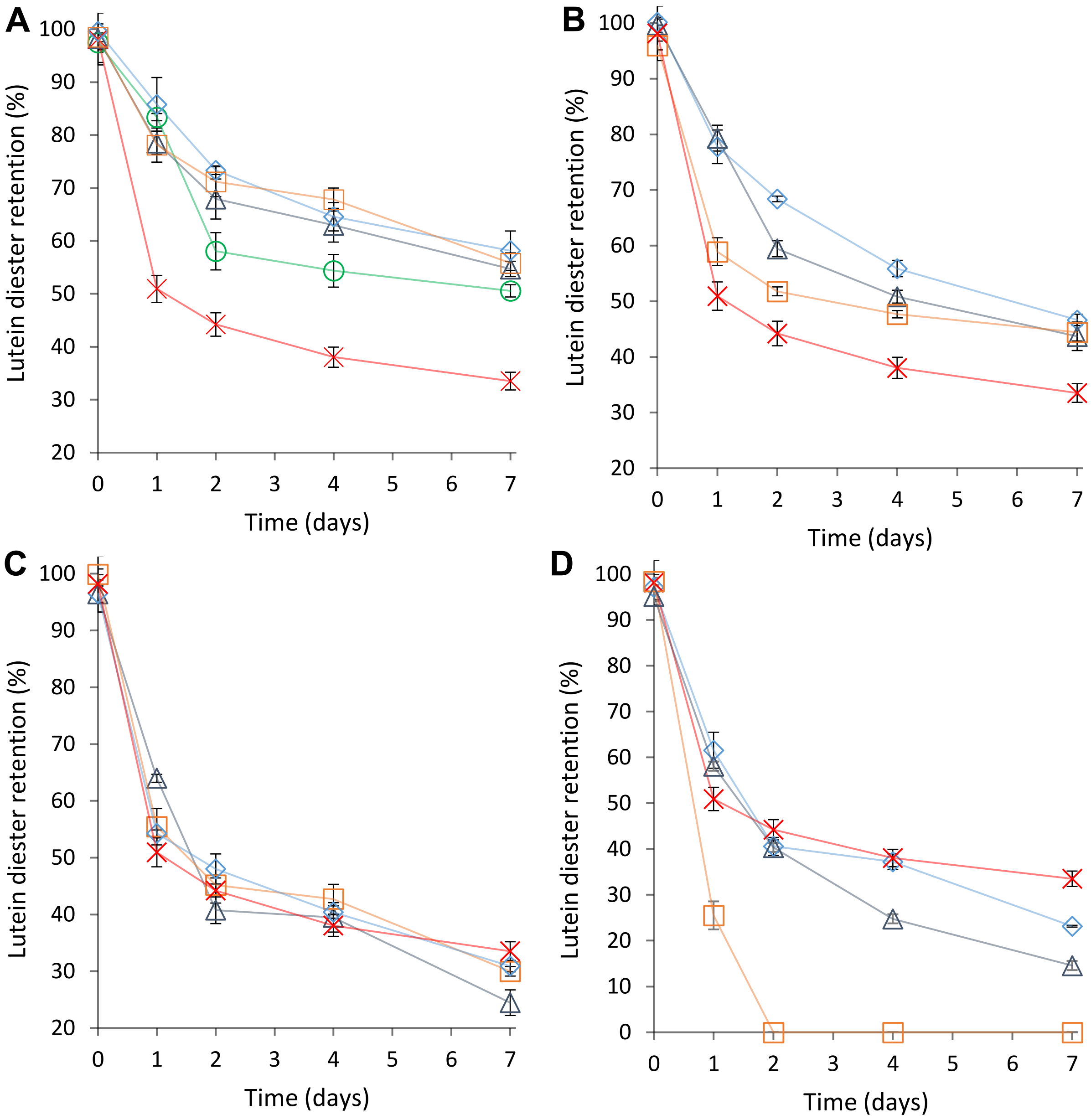

Figure S5. Retention of lutein diesters as a function of time at 60 °C. **A**: lutein di(3-methylbutanoate) (diamond), lutein di(2-methylpropanoate) (square), lutein didecanoate (triangle), lutein divalerate (circle) and lutein (cross); **B**: lutein di(2,2-dimethylpropanoate) (diamond), lutein dipalmitate (square), lutein di(pent-4-enoate) (triangle), and lutein (cross); **C**: lutein dioleate (diamond), lutein dibenzoate (square), lutein dipropanoate (triangle) and lutein (cross); **D**: lutein diacetate (diamond), lutein di(pentafluoropropanoate) (triangle), lutein diphthalate (square) and lutein (cross). Solutions of lutein diesters in ethanol (20 µM) were incubated at the elevated temperature in amber HPLC vials and under an argon atmosphere in the dark for 7 days. Experiments were carried out in triplicate.

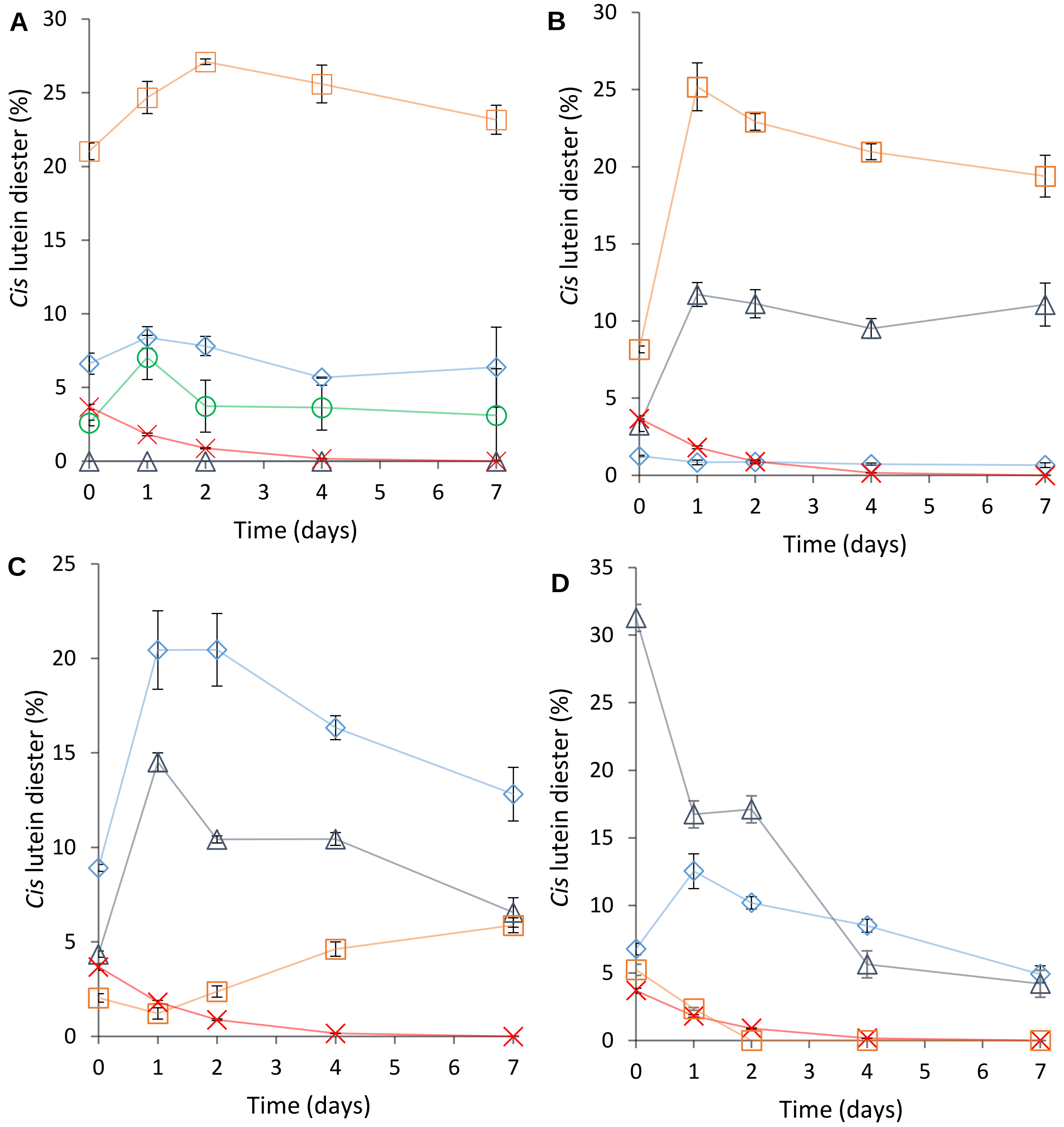

Figure S6. The content of *cis*-lutein diesters as a function of time at 60 °C. **A**: *cis*-lutein di(3-methylbutanoate) (diamond), *cis*-lutein di(2-methylpropanoate) (square), *cis*-lutein didecanoate (triangle), *cis*-lutein divalerate (circle) and *cis*-lutein (cross); **B**: *cis*-lutein di(2,2-dimethylpropanoate) (diamond), *cis*-lutein dipalmitate (square), *cis*-lutein di(pent-4-enoate) (triangle), and *cis*-lutein (cross); **C**: *cis*-lutein dioleate (diamond), *cis*-lutein dibenzoate (square), *cis*-lutein dipropanoate (triangle) and *cis*-lutein (cross); **D**: *cis*-lutein diacetate (diamond), *cis*-lutein di(pentafluoropropanoate) (square), *cis*-lutein diphthalate (triangle) and *cis*-lutein (cross). Experimental details are the same as in Figure S5.

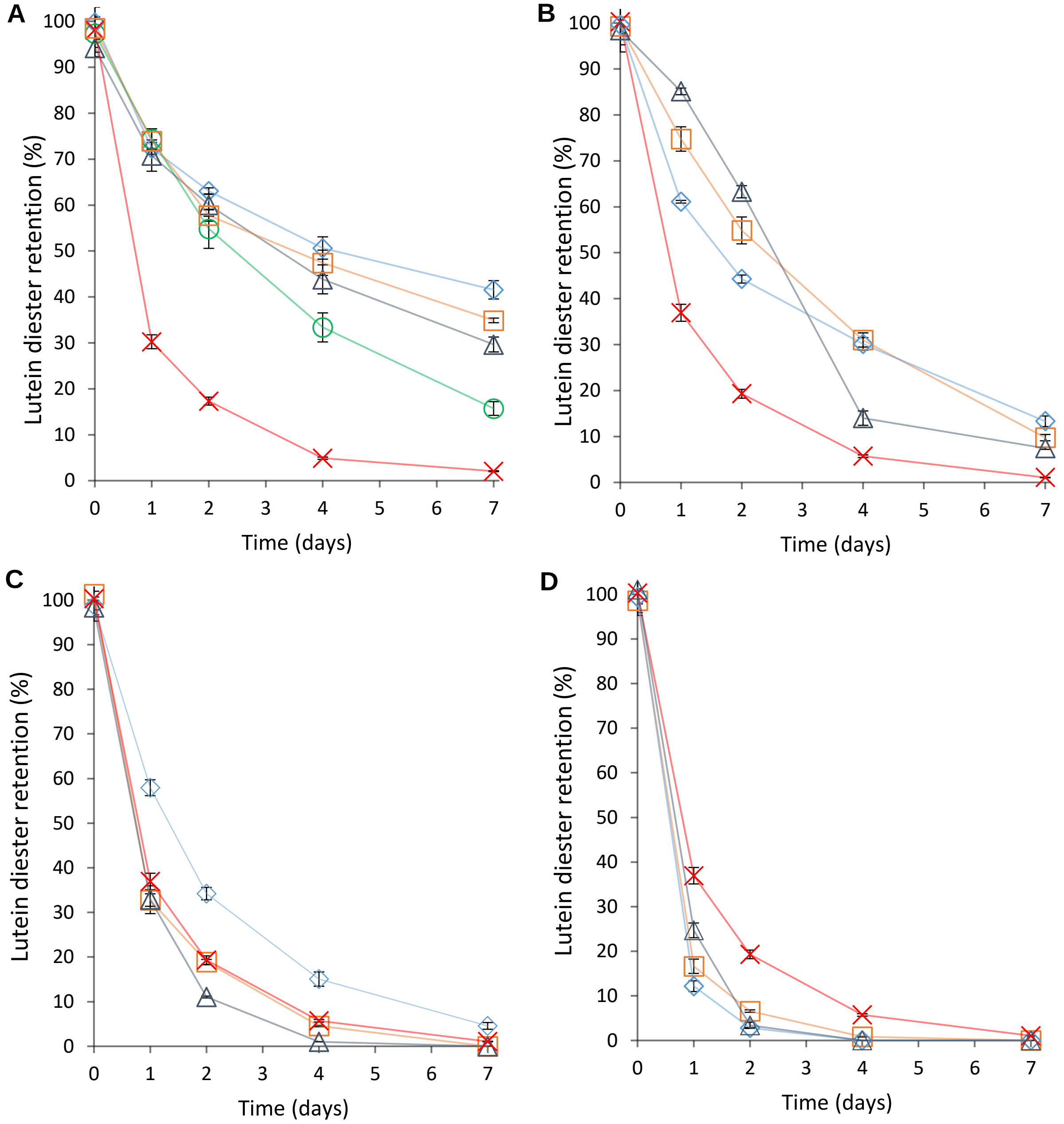

Figure S7. Retention of lutein diesters as a function of time under UV-A (365 nm) illumination: **A**: lutein di(2,2,-dimethylpropanoate) (diamond), lutein di(2-methylpropanoate) (square), lutein dipalmitate (triangle), lutein divalerate (circle) and lutein (cross); **B**: lutein di(3-methylbutanoate) (diamond), lutein di(pent-4-enoate) (square), lutein diphthalate (triangle) and lutein (cross); **C**: lutein didecanoate (diamond), lutein dibenzoate (square), lutein dioleate (triangle) and lutein (cross); **D**: lutein dipropanoate (diamond), lutein diacetate (square), lutein di(pentafluoropropanoate) (triangle) and lutein (cross). Solutions of lutein diesters in ethanol (20 µM) were incubated under UV-A light (15 cm from the light source) in clear glass HPLC vials and under an argon atmosphere for 7 days. Experiments were carried out in triplicate.

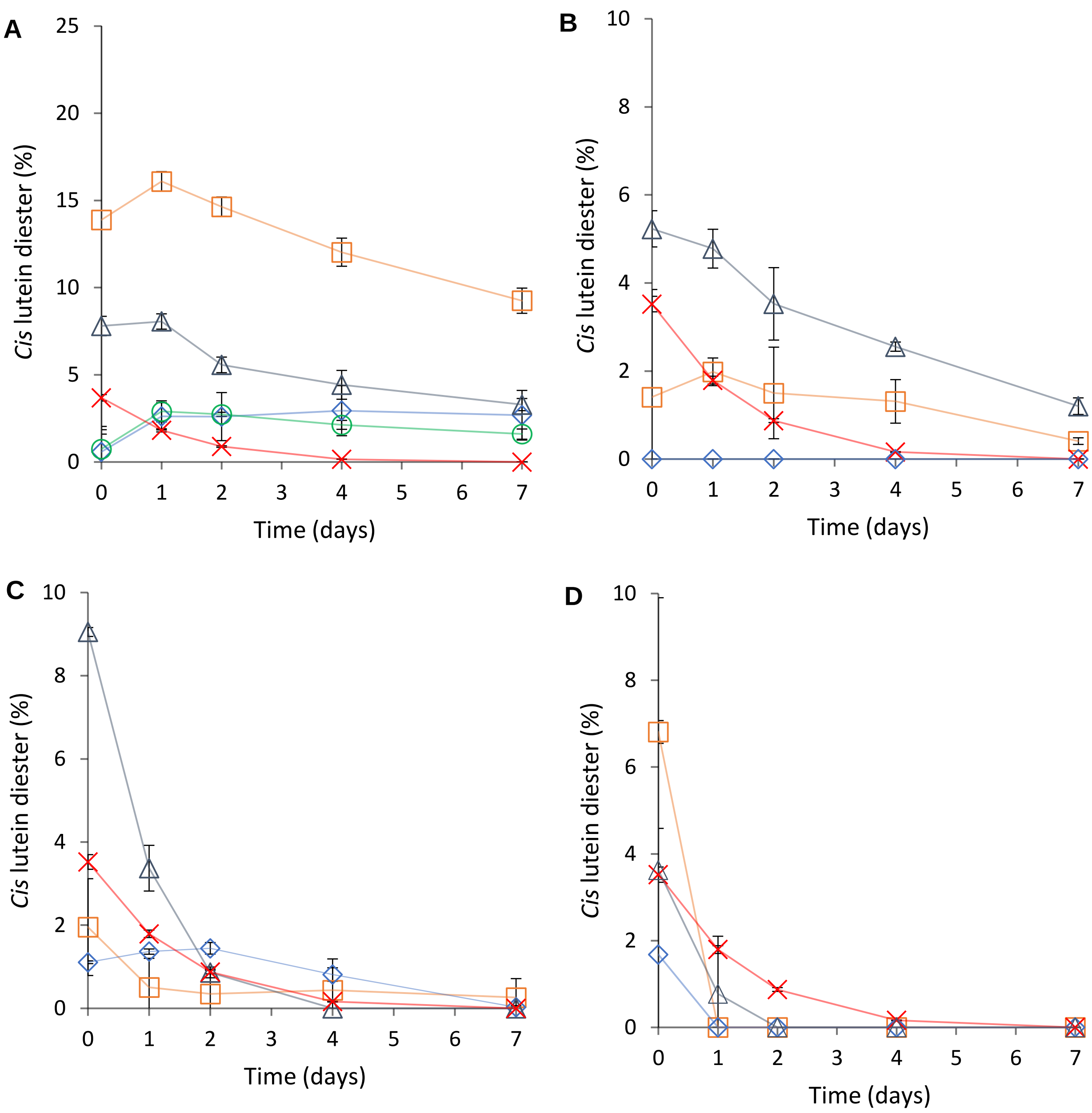

Figure S8. The content of *cis*-lutein diesters as a function of time under UV-A (365 nm) illumination: **A**: *cis*-lutein di(2,2,-dimethylpropanoate) (diamond), *cis*-lutein di(2-methylpropanoate) (square), *cis*-lutein dipalmitate (triangle), *cis*-lutein divalerate (circle) and *cis*-lutein (cross); **B**: *cis*-lutein di(3methylbutanoate) (diamond), *cis*-lutein di(pent-4-enoate) (square), *cis*-lutein diphthalate (triangle) and *cis*-lutein (cross); **C**: *cis*-lutein didecanoate (diamond), cis-lutein dibenzoate (square), *cis*-lutein dioleate (triangle) and *cis*-lutein (cross); **D**: *cis*-lutein dipropanoate (diamond), *cis*-lutein diacetate (square), *cis*-lutein di(pentafluoropropanoate) (triangle) and *cis*-lutein (cross). Experimental details are the same as in Figure S7.

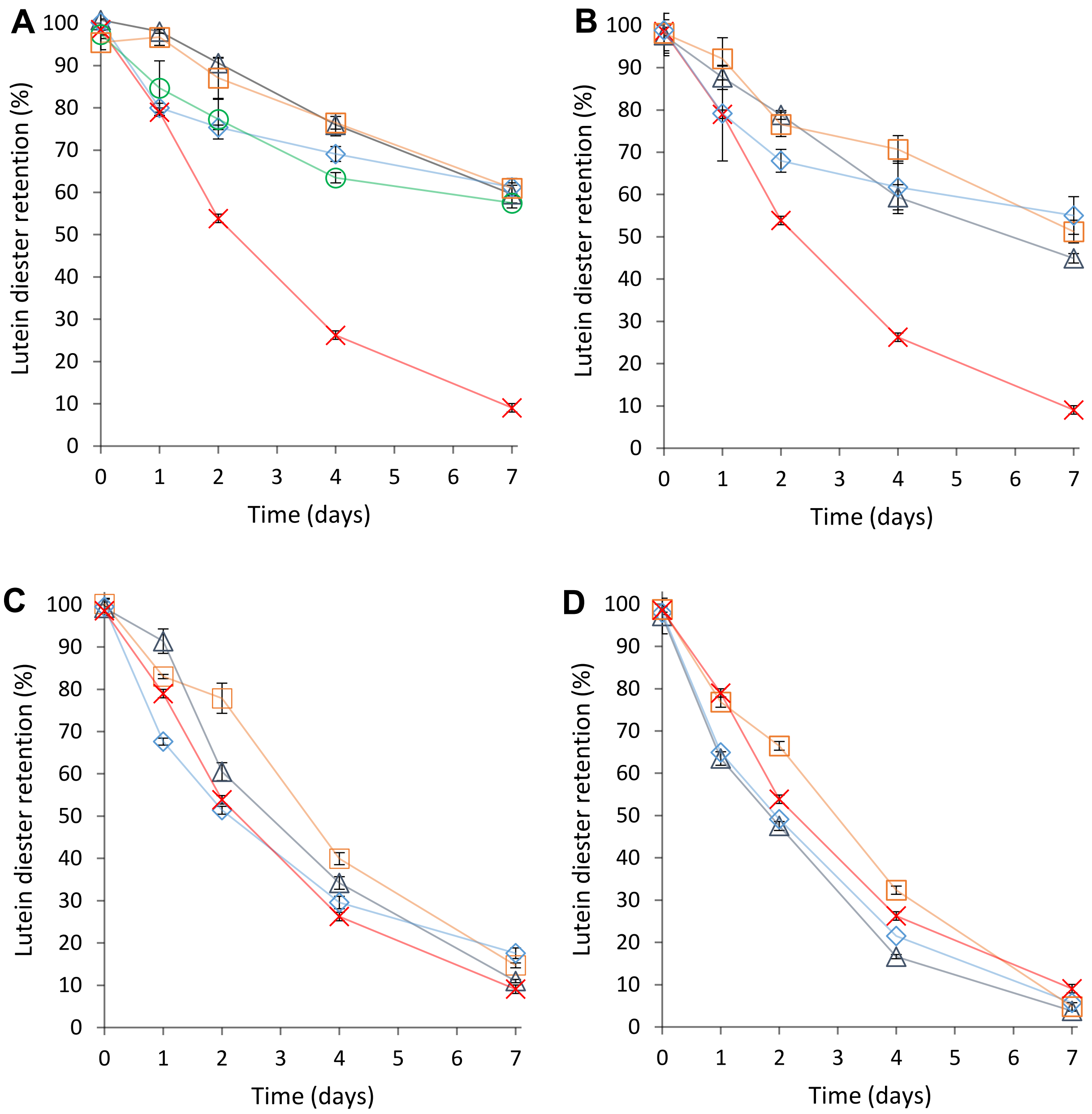

Figure S9. Retention of lutein diesters as a function of time in the presence of 0.15% $H_2O_{2(aq)}$: **A**: lutein dibenzoate (diamond), lutein di(2-methylpropanoate) (square), lutein di(2,2-dimethylpropanoate) (triangle), lutein diphthalate (circle) and lutein (cross); **B**: lutein di(pentafluoropropanoate) (diamond), lutein di(3-methylbutanoate) (square), lutein dioleate (triangle), and lutein (cross); **C**: lutein dipalmitate (diamond), lutein divalerate (square), lutein didecanoate (triangle) and lutein (cross) **D**: lutein dipropanoate (diamond), lutein di(pent-4-enoate) (square), lutein diacetate (triangle) and lutein (cross). The mixtures were incubated in amber HPLC vials under an argon atmosphere at 22 °C for 7 days. Experiments were carried out in triplicate.

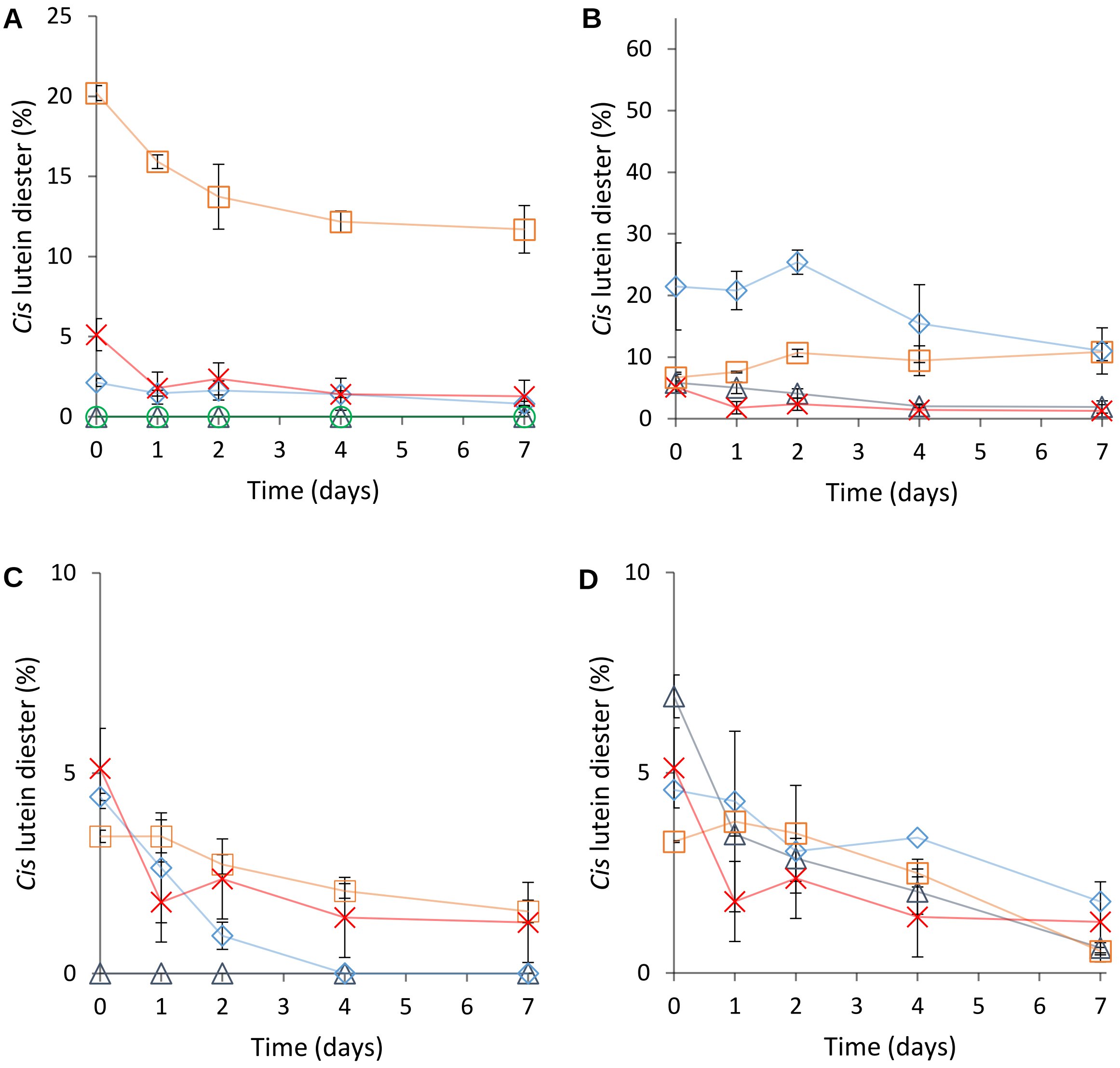

Figure S10. The content of *cis*-lutein diesters as a function of time in the presence of 0.15% $H_2O_{2(aq)}$: **A**: *cis*-lutein dibenzoate (diamond), *cis*-lutein di(2-methylpropanoate) (square), *cis*-lutein di(2,2-dimethylpropanoate) (triangle), *cis*-lutein diphthalate (circle) and *cis*-lutein (cross); **B**: *cis*-lutein di(pentafluoropropanoate) (diamond), *cis*-lutein di(3-methylbutanoate) (square), *cis*-lutein dioleate (triangle), and *cis*-lutein (cross); **C**: *cis*-lutein dipalmitate (diamond), *cis*-lutein divalerate (square), *cis*-lutein didecanoate (triangle) and *cis*-lutein (cross) **D**: *cis*-lutein dipropanoate (diamond), *cis*-lutein di(pent-4-enoate) (square), *cis*-lutein diacetate (triangle) and *cis*-lutein (cross). Experimental details are the same as in Figure S9.

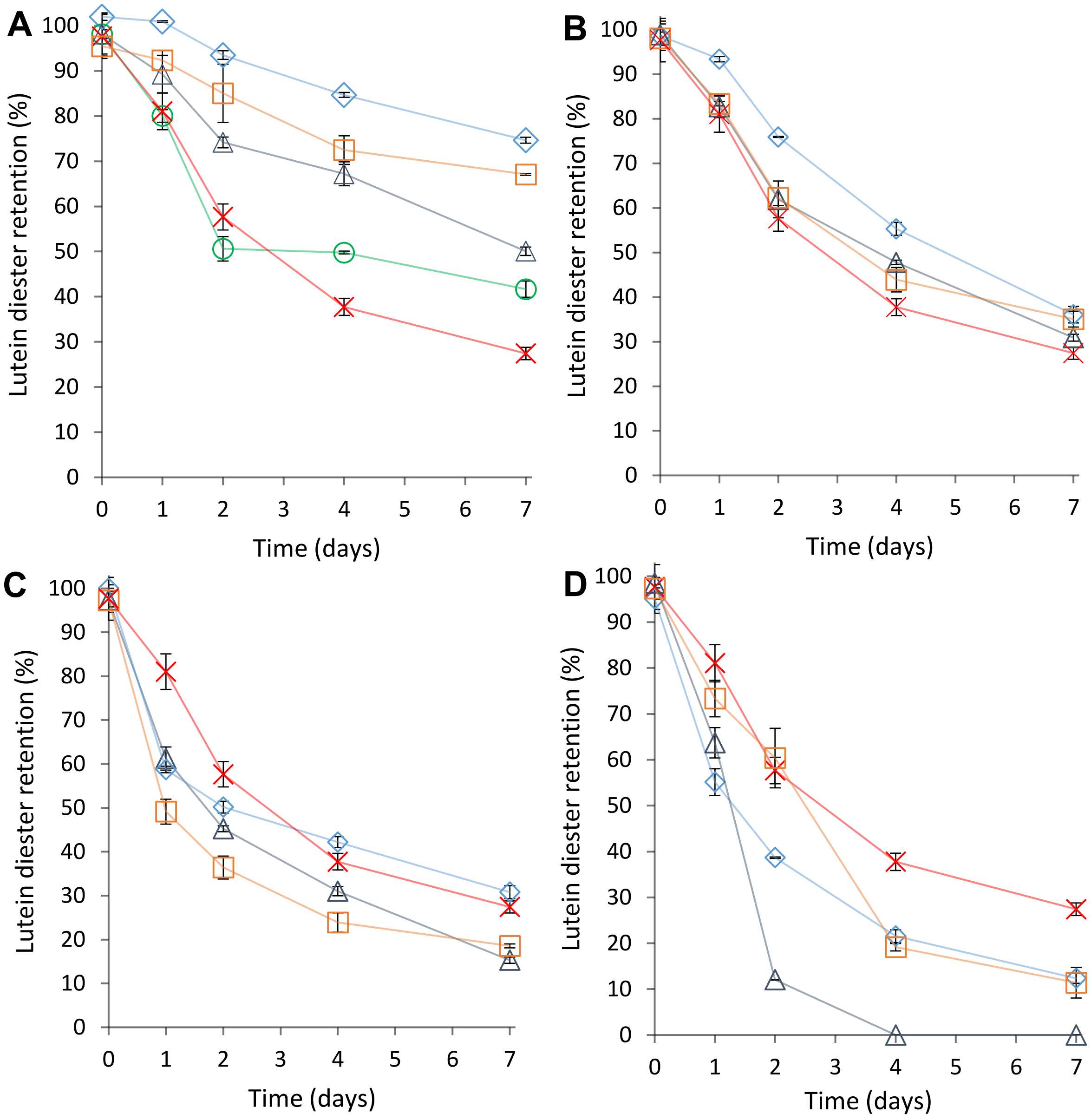

Figure S11. Retention of lutein diesters as a function of time in the presence of 5% (v/v) of 200 mM ammonium formate buffer (pH = 2): **A**: lutein di(2,2-dimethylpropanoate) (diamond), lutein di(2-methylpropanoate) (square), lutein di(3-methylbutanoate) (triangle), lutein dipalmitate (circle) and lutein (cross); **B**: lutein divalerate (diamond), lutein didecanoate (square), lutein di(pent-4-enoate) (triangle) and lutein (cross); **C**: lutein dibenzoate (diamond), lutein dioleate (square), lutein diacetate (triangle) and lutein (cross); **D**: lutein dipropanoate (diamond), lutein di(pentafluoropropanoate) (square), lutein diphthalate (triangle) and lutein (cross). The mixtures were incubated in amber HPLC vials under an argon atmosphere at 22 °C for 7 days. Experiments were carried out in triplicate.

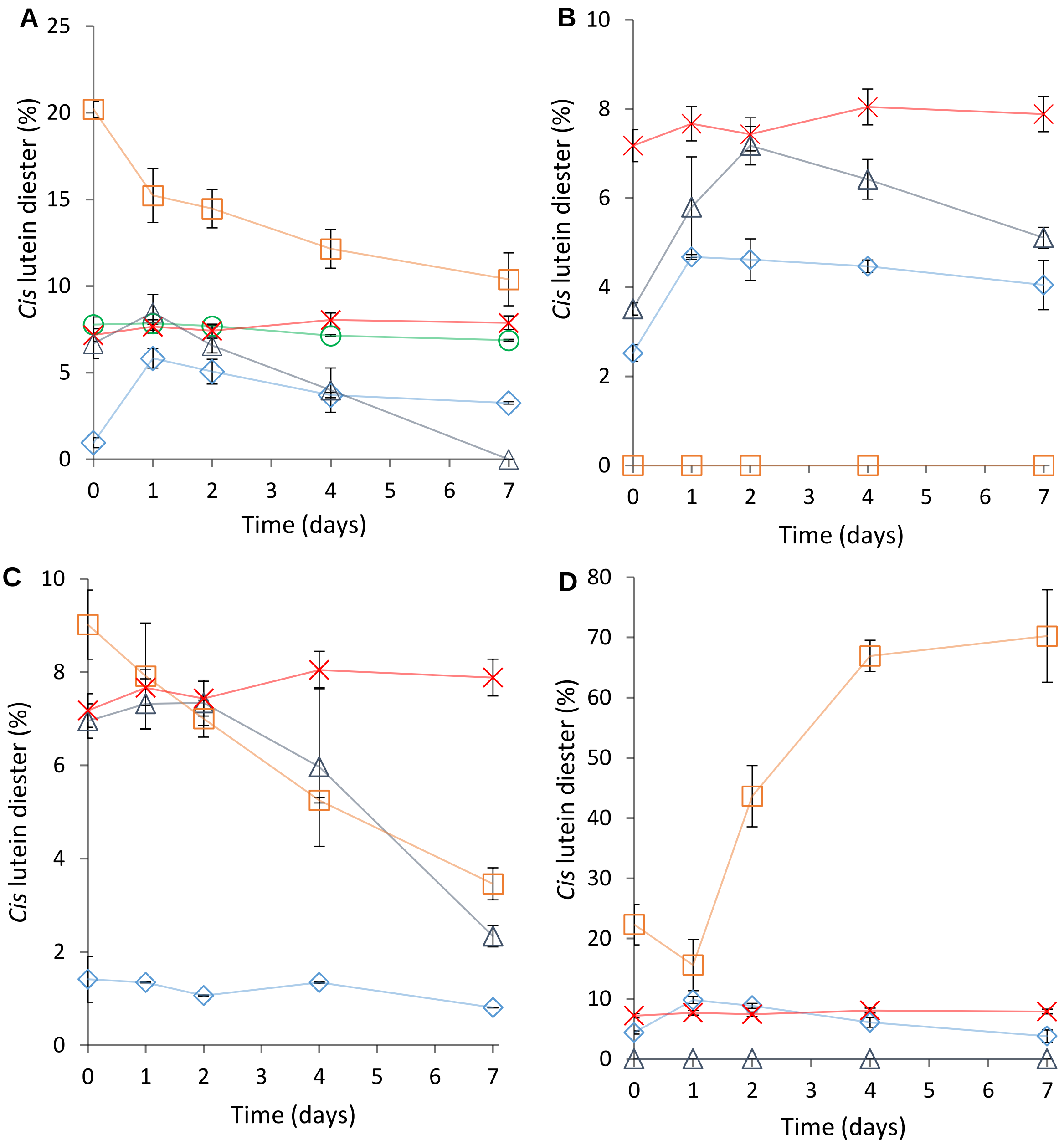

Figure S12. The content of *cis*-lutein diesters as a function of time in the presence of 5% (v/v) of 200 mM ammonium formate buffer (pH = 2): **A**: *cis*-lutein di(2,2-dimethylpropanoate) (diamond), *cis*-lutein di(2-methylpropanoate) (square), *cis*-lutein di(3-methylbutanoate) (triangle), *cis*-lutein dipalmitate (circle) and *cis*-lutein (cross); **B**: *cis*-lutein divalerate (diamond), *cis*-lutein didecanoate (square), *cis*-lutein di(pent-4-enoate) (triangle) and *cis*-lutein (cross); **C**: *cis*-lutein dibenzoate (diamond), *cis*-lutein dioleate (square), *cis*-lutein diacetate (triangle) and *cis*-lutein (cross); **D**: *cis*-lutein dipropanoate (diamond), *cis*-lutein di(pentafluoropropanoate) (square), *cis*-lutein diphthalate (triangle) and *cis*-lutein (cross). Experimental details are the same as in Figure S11.

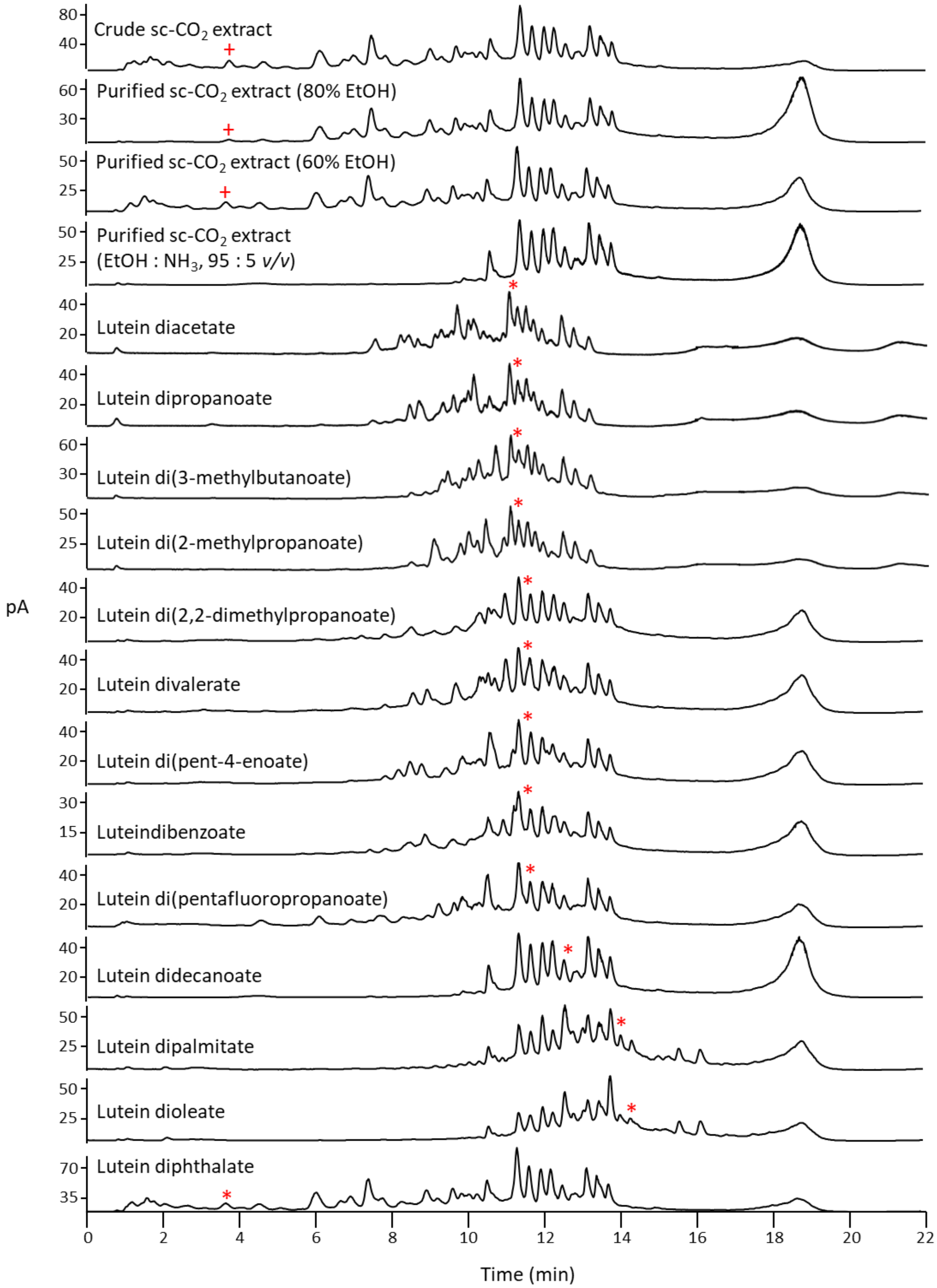

Figure S13. HPLC-CAD chromatograms of raw and purified sc-CO_2 extracts of Japanese knotweed green leaves and synthesized lutein diesters (from sc-CO_2 extract) after SPE purification. The corresponding lutein diesters are marked with a red asterisk (*) while free lutein with a plus symbol (+). See Experimental for details.

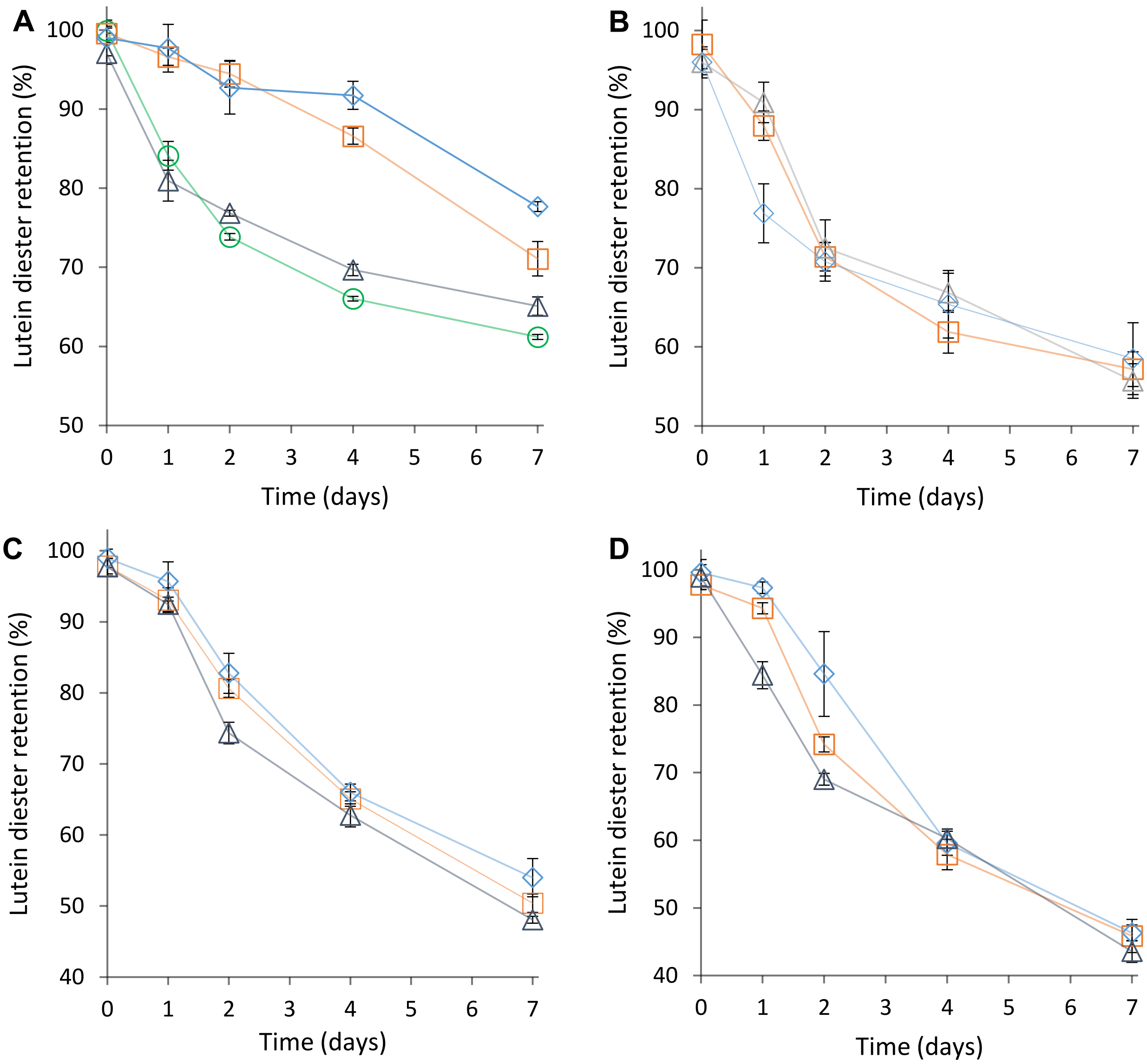

Figure S14. Retention of lutein diesters prepared from the Japanese knotweed green leaf extract as a function of time at 60 °C: **A**: lutein di(2-methylpropanoate) (diamond), lutein di(3-methylbutanoate) (square), lutein didecanoate (triangle) and lutein diphthalate (circle); **B**: lutein dipalmitate (diamond), lutein di(2,2-dimethylpropanoate) (square) and lutein divalerate (triangle); **C**: lutein dipropanoate (diamond), lutein diacetate (square) and lutein di(pent-4-enoate) (triangle); **D**: lutein dioleate (diamond), lutein dibenzoate (square) and lutein di(pentafluoropropanoate) (triangle). Solutions of lutein diesters in ethanol (20 µM) were incubated at the elevated temperature in amber HPLC vials and under an argon atmosphere in the dark for 7 days. Experiments were carried out in triplicate.

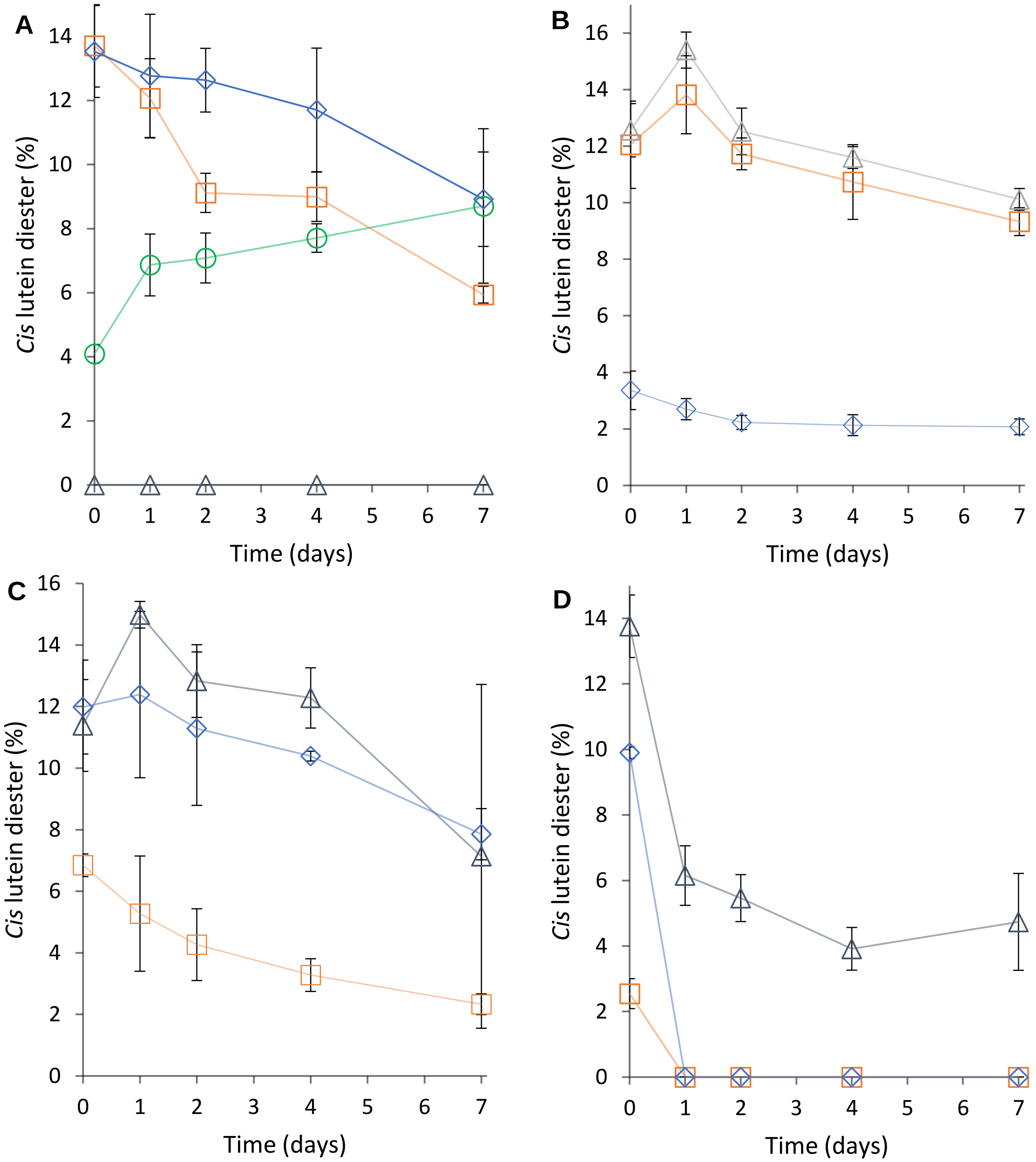

Figure S15. The content of *cis*-lutein diesters from Japanese knotweed green leaf extract as a function of time at 60 °C: **A**: *cis*-lutein di(2-methylpropanoate) (diamond), *cis*-lutein di(3-methylbutanoate) (square), *cis*-lutein didecanoate (triangle) and *cis*-lutein diphthalate (circle); **B**: *cis*-lutein dipalmitate (diamond), *cis*-lutein di(2,2-dimethylpropanoate) (square) and *cis*-lutein divalerate (triangle); **C**: *cis*-lutein dipropanoate (diamond), *cis*-lutein diacetate (square) and *cis*-lutein di(pent-4-enoate) (triangle); **D**: *cis*-lutein dioleate (diamond), *cis*-lutein dibenzoate (square) and *cis*-lutein di(pentafluoropropanoate) (triangle). Experimental details are the same as in Figure S14.

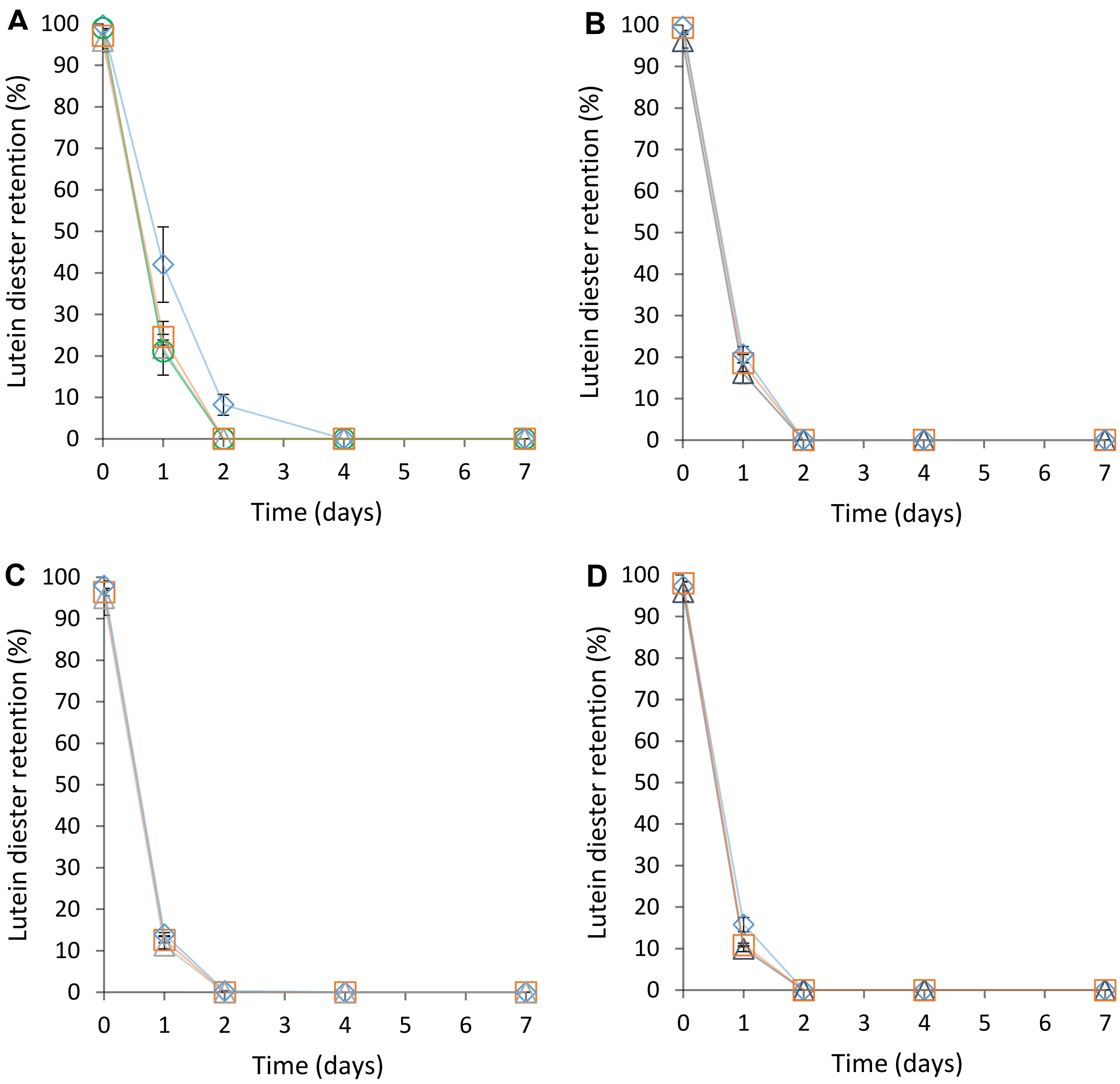

Figure S16. Retention of lutein diesters prepared from the Japanese knotweed green leaf extract as a function of time under UV-A (365 nm) illumination: **A**: lutein diphthalate (diamond), lutein di(pentafluoropropanoate) (square), lutein di(2,2,-dimethylpropanoate) (triangle) and lutein di(2-methylpropanoate) (circle); **B**: lutein di(3methylbutanoate) (diamond), lutein di(pent-4-enoate) (square) and lutein diacetate (triangle); **C**: lutein divalerate (diamond), lutein didecanoate (square), and lutein dipropanoate (triangle); **D**: lutein dipalmitate (diamond), lutein dioleate (square) and lutein dibenzoate (triangle). Solutions of lutein diesters in ethanol (20 µM) were incubated under UV-A light (15 cm from the light source) in clear glass HPLC vials and under an argon atmosphere for 7 days. Experiments were carried out in triplicate.

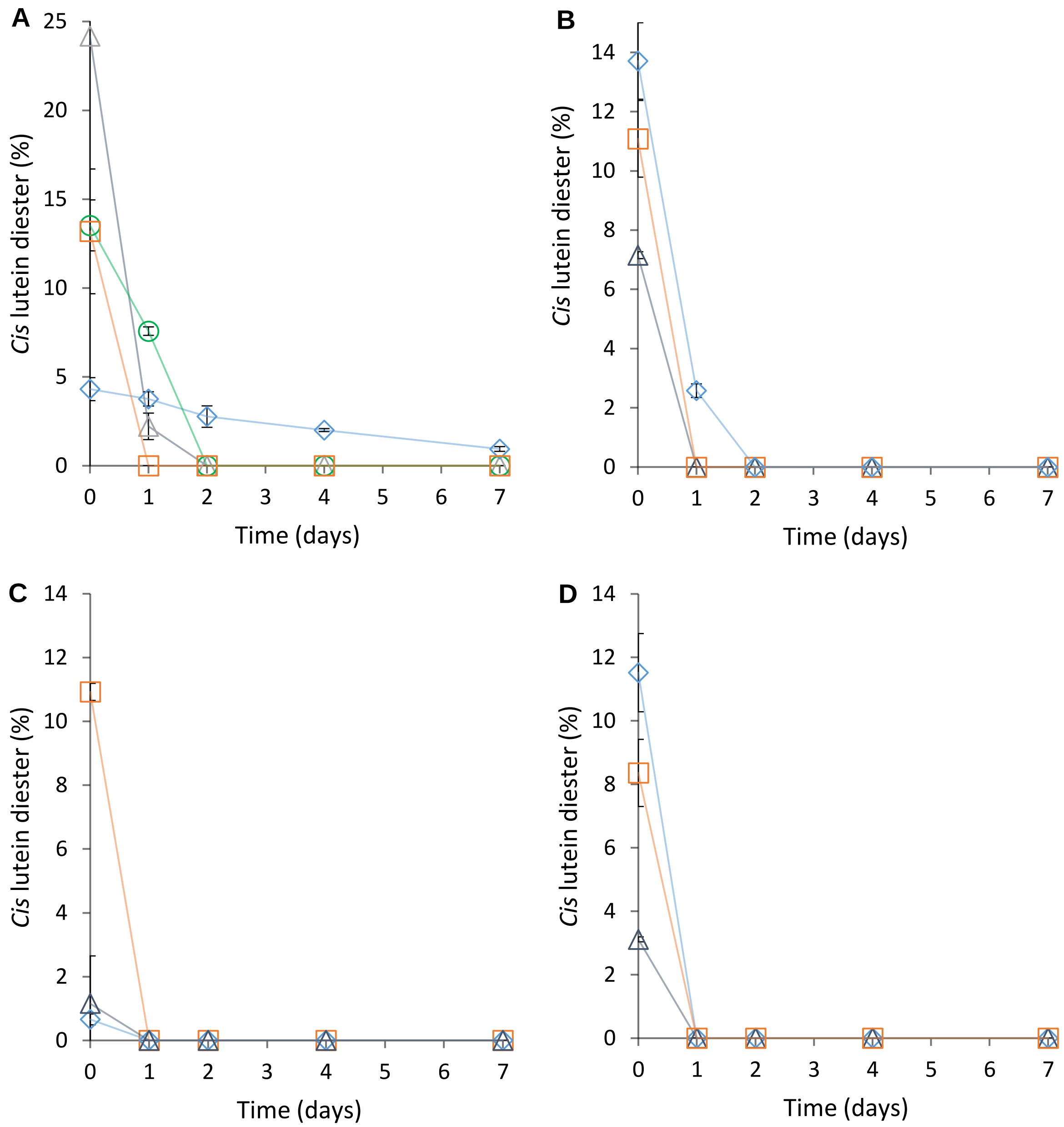

Figure S17. The content of *cis*-lutein diesters from Japanese knotweed green leaf extract as a function of time under UV-A (365 nm) illumination: **A** *cis*-lutein diphthalate (diamond), *cis*-lutein di(pentafluoropropanoate) (square), *cis*-lutein di(2,2,-dimethylpropanoate) (triangle) and *cis*-lutein di(2-methylpropanoate) (circle); **B**: *cis*-lutein di(3methylbutanoate) (diamond), *cis*-lutein di(pent-4-enoate) (square) and *cis*-lutein diacetate (triangle); **C**: *cis*-lutein divalerate (diamond), *cis*-lutein didecanoate (square), and *cis*-lutein dipropanoate (triangle); **D**: *cis*-lutein dipalmitate (diamond), *cis*-lutein dioleate (square) and *cis*-lutein dibenzoate (triangle). Experimental details are the same as in Figure S16.

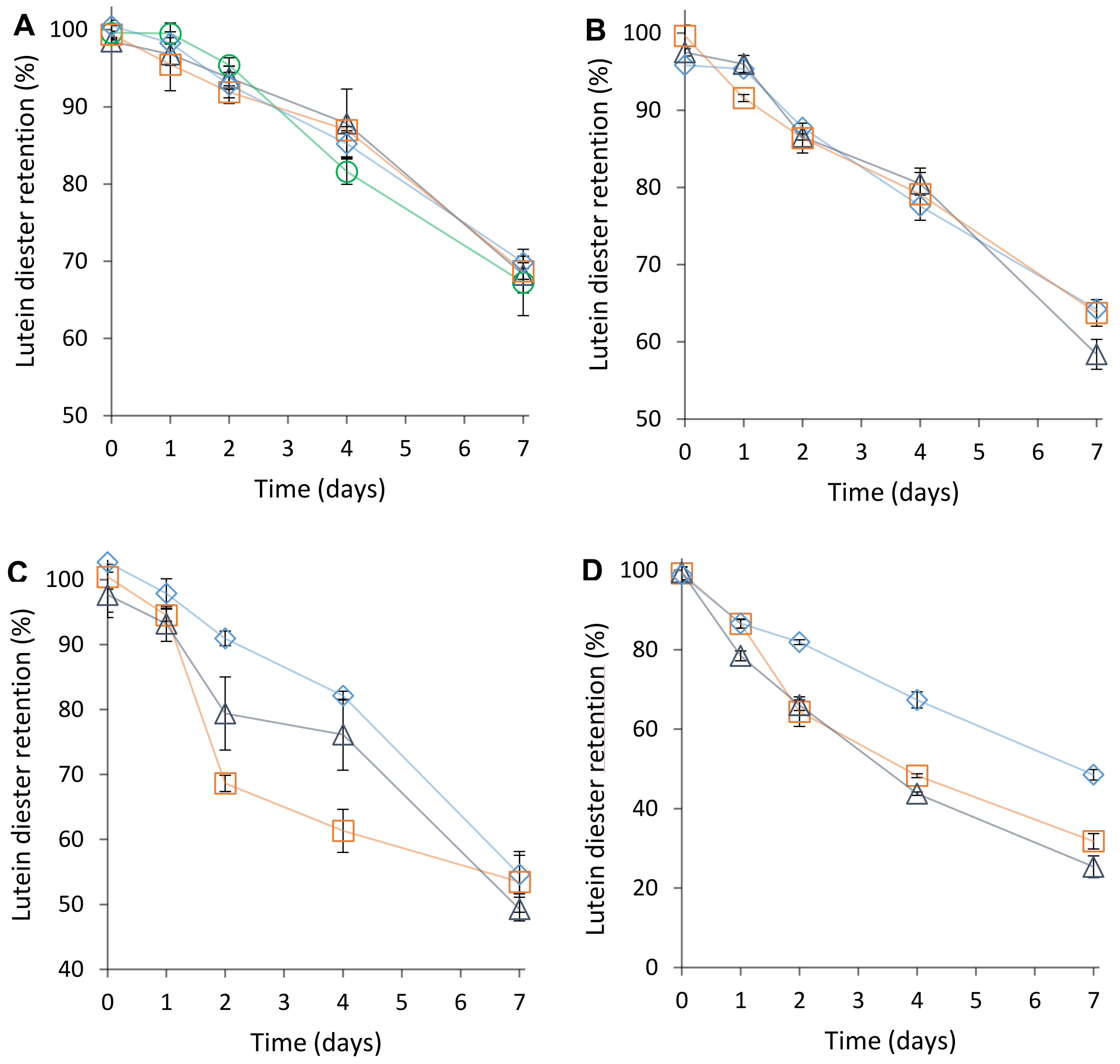

Figure S18. Retention of lutein diesters prepared from the Japanese knotweed green leaf extract as a function of time in the presence of 0.15% $H_2O_{2(aq)}$: **A**: lutein didecanoate (diamond), lutein di(pent-4-enoate) (square), lutein diacetate (triangle) and lutein divalerate (circle); **B**: lutein dipropanoate (diamond), lutein diphthalate (square) and lutein di(2,2-dimethylpropanoate) (triangle); **C**: lutein dibenzoate (diamond), lutein di(2-methylpropanoate) (square) and lutein di(3-methylbutanoate) (triangle); **D**: lutein di(pentafluoropropanoate) (diamond), lutein dipalmitate (square) and lutein dioleate (triangle). The mixtures were incubated in amber HPLC vials under an argon atmosphere at 22 °C for 7 days. Experiments were carried out in triplicate.

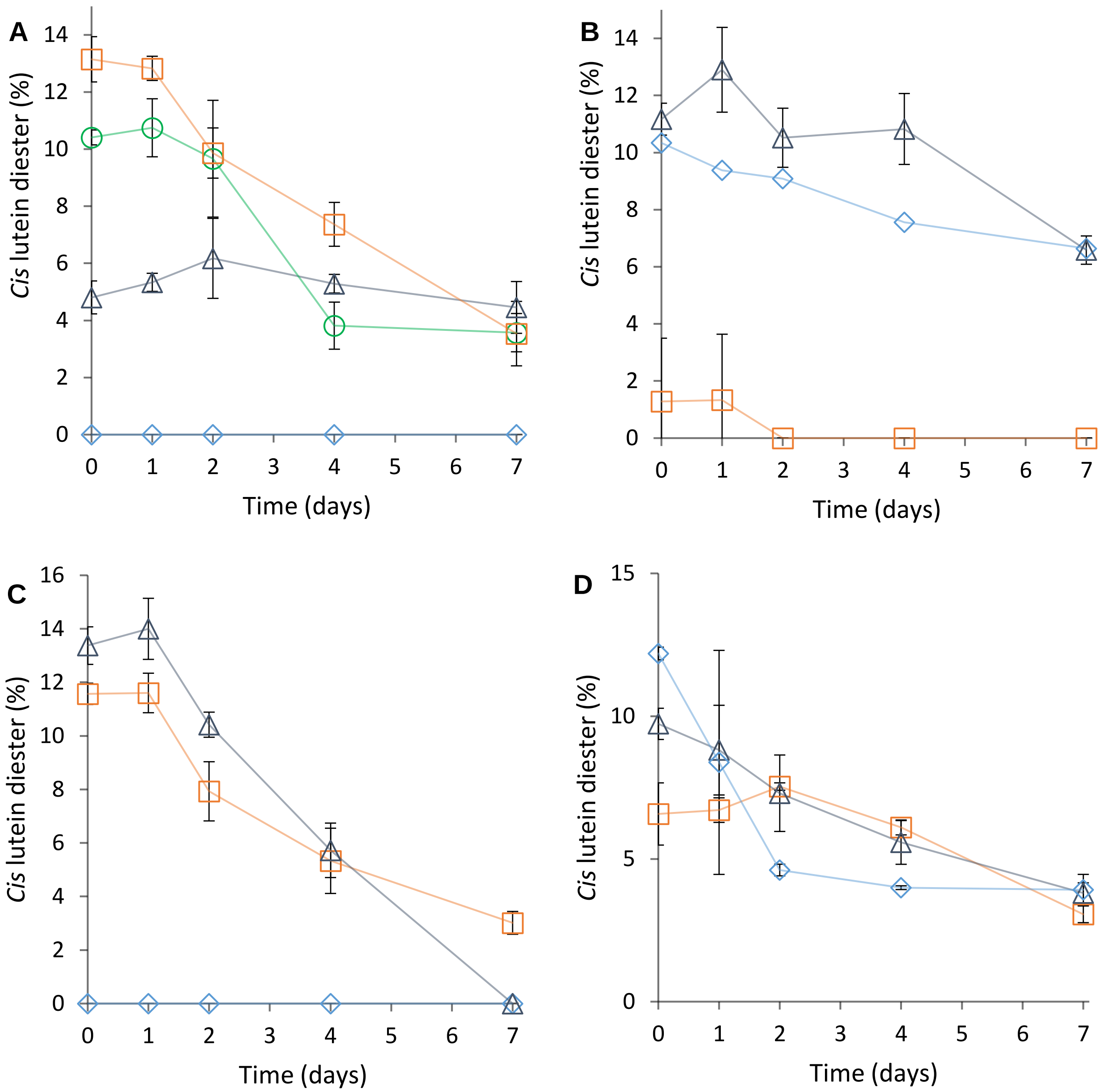

Figure S19. The content of *cis*-lutein diesters from Japanese knotweed green leaf extract as a function of time in the presence of 0.15% $H_2O_{2(aq)}$: **A**: *cis*-lutein didecanoate (diamond), *cis*-lutein di(pent-4-enoate) (square), *cis*-lutein diacetate (triangle) and *cis*-lutein divalerate (circle); **B**: *cis*-lutein dipropanoate (diamond), *cis*-lutein diphthalate (square) and *cis*-lutein di(2,2-dimethylpropanoate) (triangle); **C**: *cis*-lutein dibenzoate (diamond), *cis*-lutein di(3-methylbutanoate) (square) and *cis*-lutein di(2-methylpropanoate); **D**: *cis*-lutein di(pentafluoropropanoate) (diamond), *cis*-lutein dipalmitate (square) and *cis*-lutein dioleate (triangle). Experimental details are the same as in Figure S18.

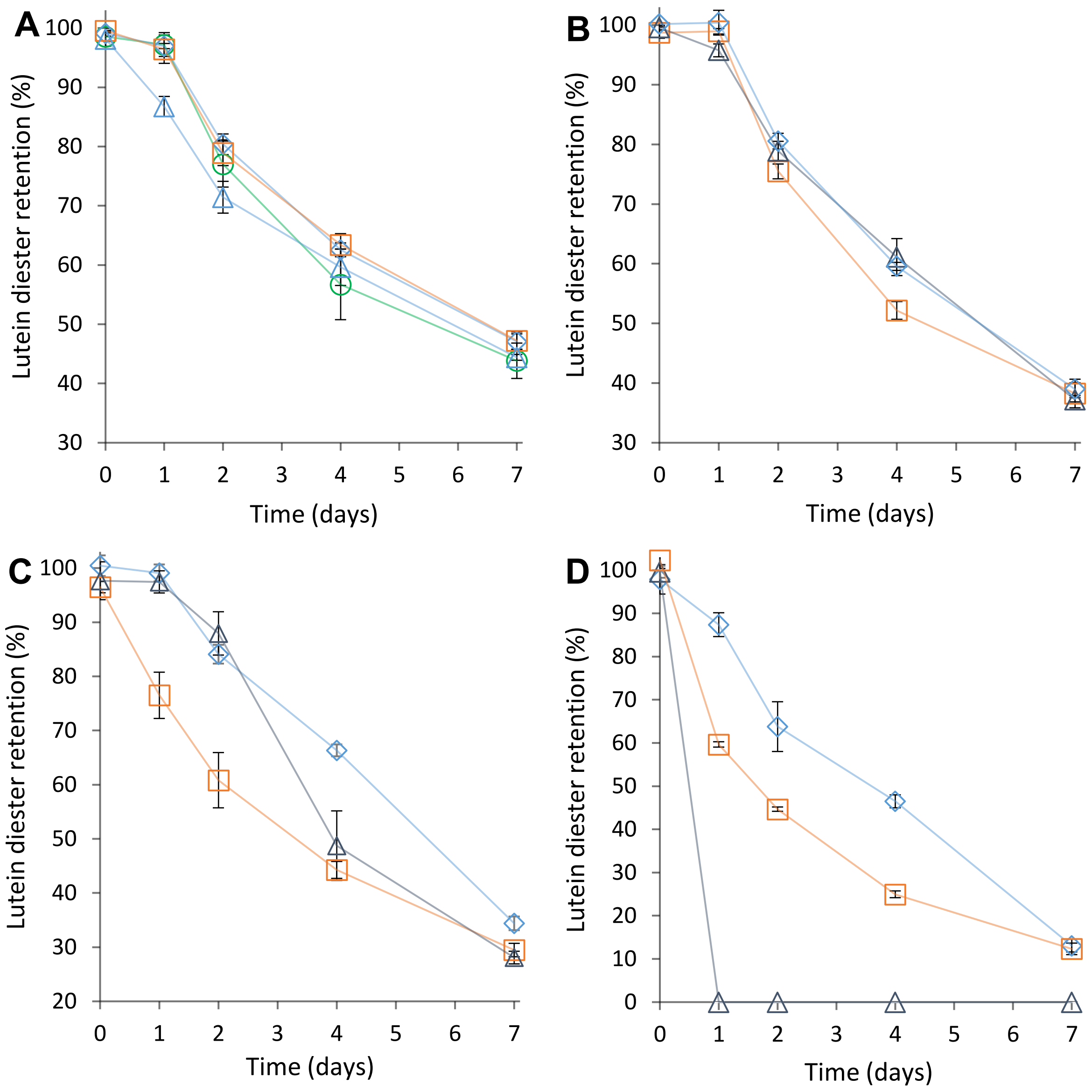

Figure S20. Retention of lutein diesters prepared from the Japanese knotweed green leaf extract as a function of time in the presence of 5% (v/v) of 200 mM ammonium formate buffer (pH = 2): **A**: lutein divalerate (diamond), lutein di(pent-4-enoate) (square), lutein didecanoate (triangle), lutein dipropanoate (circle); **B**: lutein dibenzoate (diamond), lutein diacetate (square), lutein di(2,2-dimethylpropanoate) (triangle); **C**: lutein di(3-methylbutanoate) (diamond) lutein dipalmitate (square) and lutein di(2-methylpropanoate) (triangle); **D**: lutein dioleate (diamond), lutein di(pentafluoropropanoate) (square) and lutein diphthalate (triangle). The mixtures were incubated in amber HPLC vials under an argon atmosphere at 22 °C for 7 days. Experiments were carried out in triplicate.

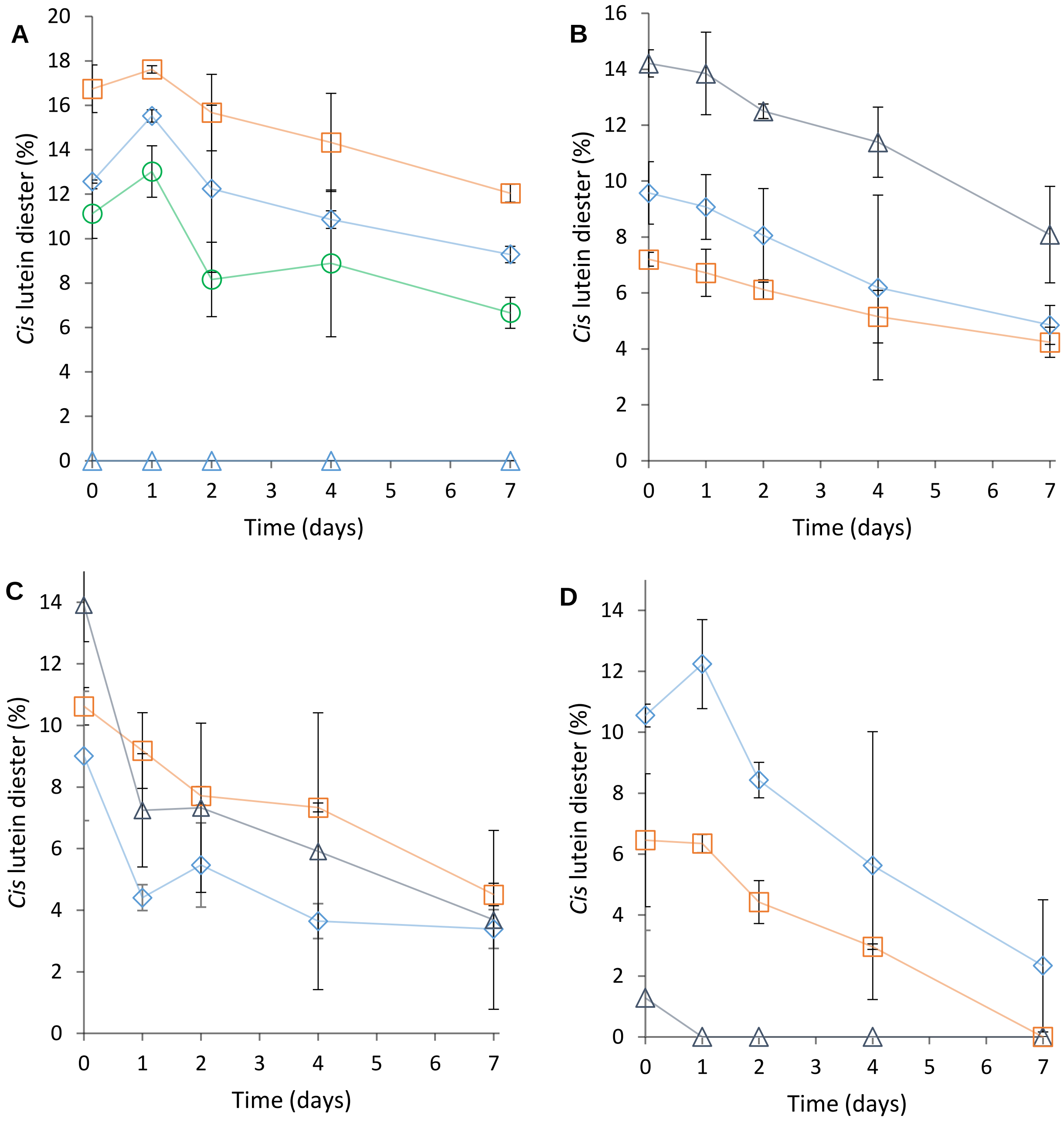

Figure S21. The content of *cis*-lutein diesters from Japanese knotweed green leaf extract as a function of time in the presence of 5% (v/v) of 200 mM ammonium formate buffer (pH = 2): **A**: *cis*-lutein divalerate (diamond), *cis*-lutein di(pent-4-enoate) (square), *cis*-lutein didecanoate (triangle), *cis*-lutein dipropanoate (circle); **B**: *cis*-lutein dibenzoate (diamond), *cis*-lutein diacetate (square), *cis*-lutein di(2,2-dimethylpropanoate) (triangle); **C**: *cis*-lutein di(3-methylbutanoate) (diamond), *cis*-lutein dipalmitate (square) and *cis*-lutein di(2-methylpropanoate) (triangle); **D** *cis*-lutein dioleate (diamond), *cis*-lutein di(pentafluoropropanoate) (square) and *cis*-lutein diphthalate (triangle). Experimental details are the same as in Figure S20.

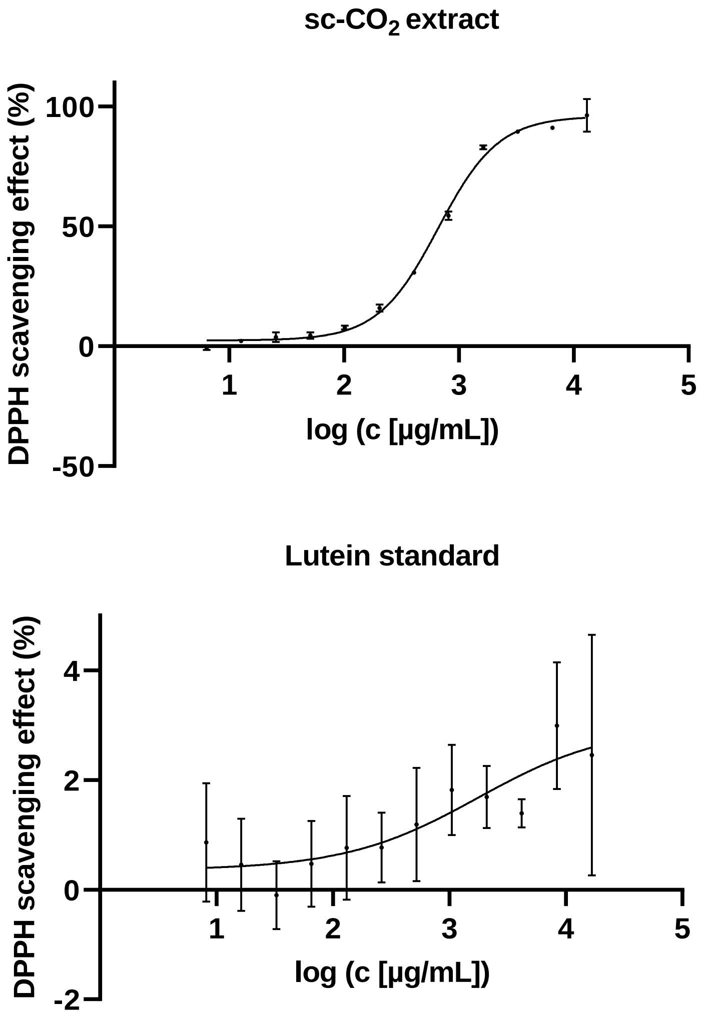

Figure S22. The antioxidant activity of solutions (n = 3) as a function of free lutein concentration: solution of sc-CO_2 extract of Japanese knotweed green leaves (top) and lutein standard solution (bottom). See Experimental for details.

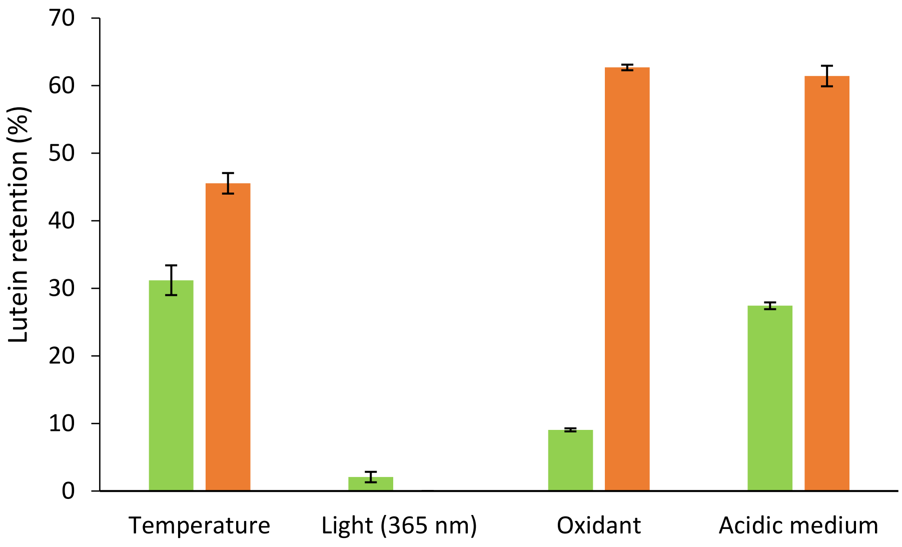

Figure S23. Retention of lutein in solution (green columns) and lutein in Japanese knotweed leaf extract matrix (orange columns) after 7 days of exposure to different stress conditions: increased temperature – 60 °C, light – 366 nm, oxidant – H_2O_2, and an acidic medium. Experiments were carried out in triplicate. Exact conditions are described in Experimental.

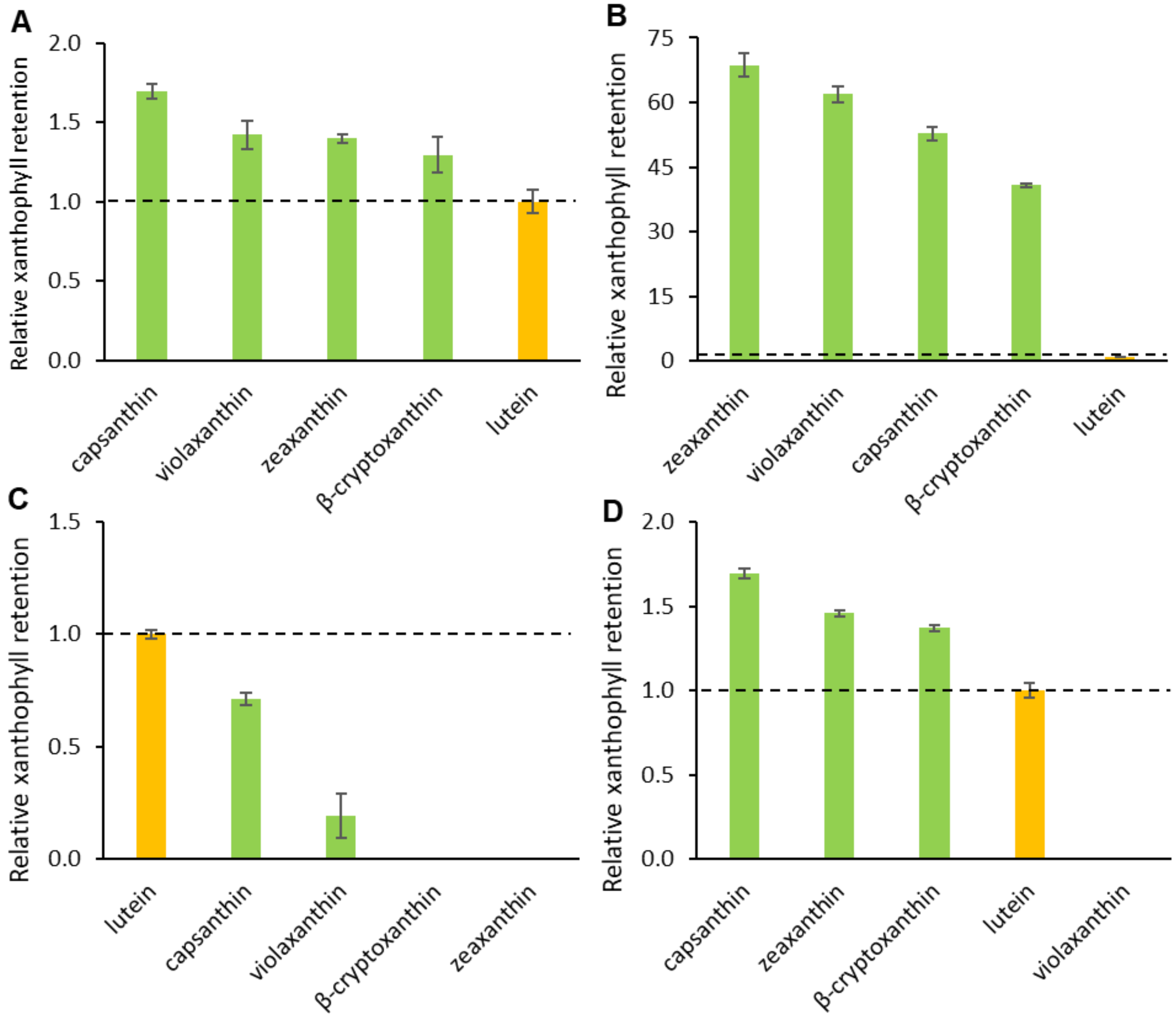

Figure S24. Retention of free xanthophylls after 7 days of exposure to different stress conditions: increased temperature – 60 °C (A), light – 366 nm (B), oxidant – H_2O_2 (C), and an acidic medium (D). Experiments were carried out in triplicate. Exact conditions are described in Experimental.

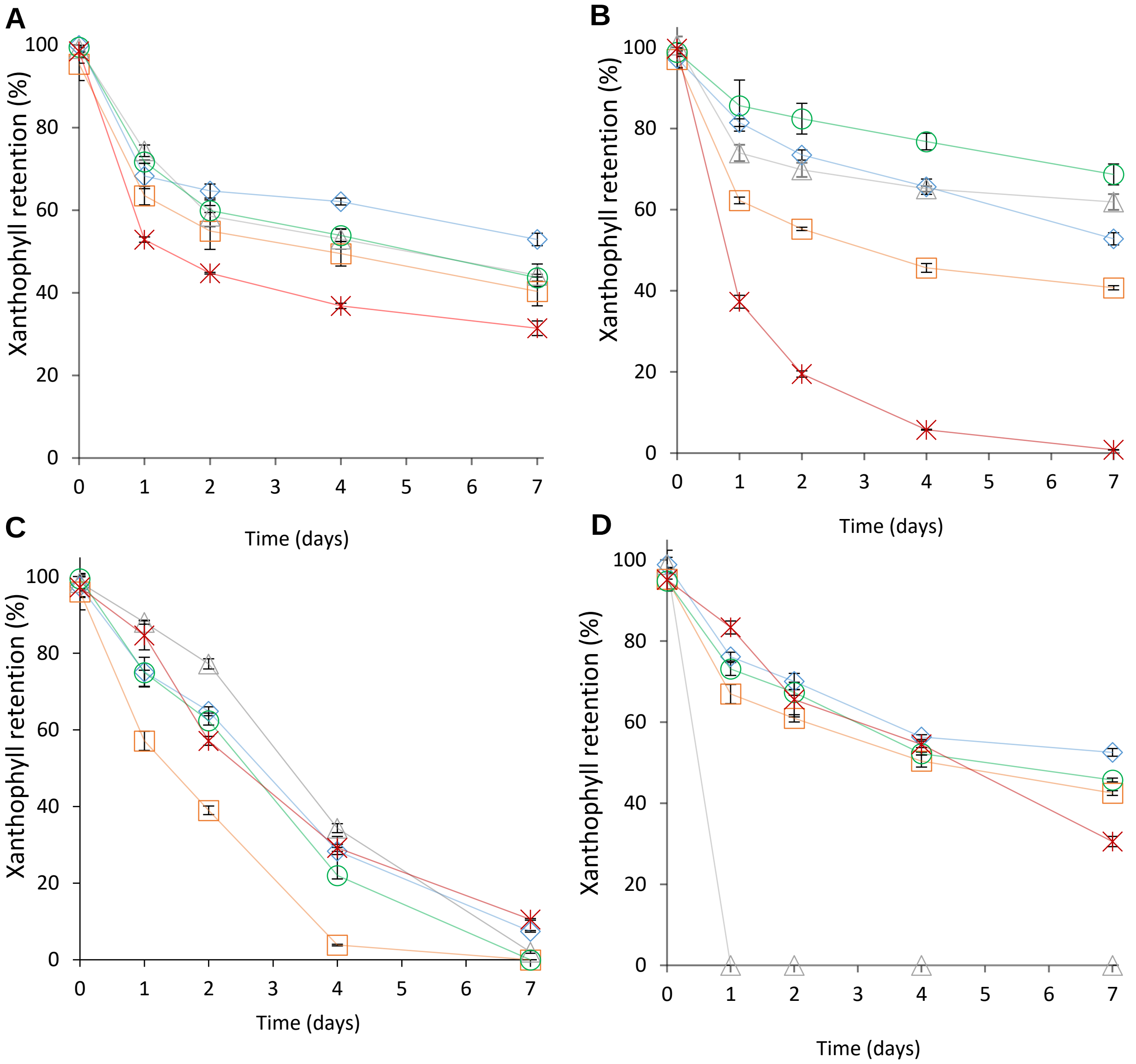

Figure S25. Retention of xanthophylls during the 7-day exposure of samples to different stress conditions: elevated temperature – 60 °C (A), light – 366 nm (B), oxidant – H_2O_2 (C) and an acidic medium (D); capsanthin (diamond), β-cryptoxanthin (square), violaxanthin (triangle), zeaxanthin (circle) and lutein (asterisk). Error bars depict the standard deviation of analytical measurements. Experiments were carried out in triplicate. Exact conditions are described in Experimental.

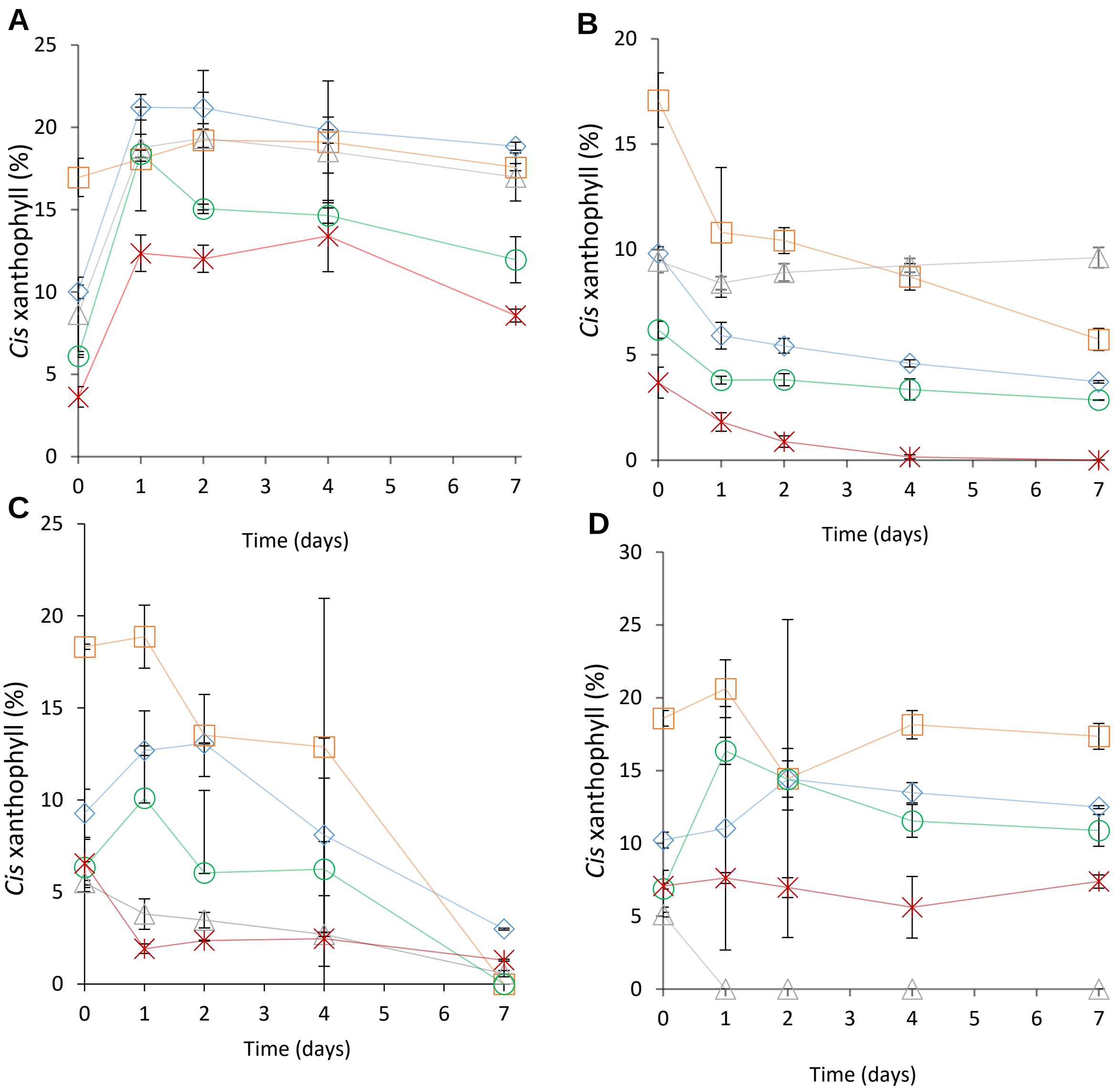

Figure S26. The content of *cis*-xanthophylls during the 7-day exposure of samples to different stress conditions: elevated temperature – 60 °C (A), light – 366 nm (B), oxidant – H_2O_2 (C) and an acidic medium (D); *cis*-capsanthin (diamond), *cis*-β-cryptoxanthin (square), *cis*-violaxanthin (triangle), *cis*-zeaxanthin (circle) and *cis*-lutein (asterisk). Error bars depict the standard deviation of analytical measurements. Experiments were carried out in triplicate. Exact conditions are described in Experimental.

References

1. Britton, G., **1995**. UV/Visible Spectroscopy. In: Carotenoids: Volume 1B: Spectroscopy. Britton, G., Liaaen-Jensen, S., Pfander H.,Birkhäuser Verlag Basel, Switzerland, pp.13–62.

2. Jug, U.; Naumoska, K.; Vovk, I. (−)-Epicatechin—An Important Contributor to the Antioxidant Activity of Japanese Knotweed Rhizome Bark Extract as Determined by Antioxidant Activity-Guided Fractionation. *Antioxidants* **2021**, *10*, 133, DOI:10.3390/antiox10010133.

2. Aparicio-Ruiz, R.; Mínguez-Mosquera, M. I.; Gandul-Rojas, B. Thermal Degradation Kinetics of Lutein, β-Carotene and β-Cryptoxanthin in Virgin Olive Oils. *J. Food Compos. Anal.* **2011**, *24*, 811–820, DOI: 10.1016/j.jfca.2011.04.009.

3. Bockuviene, A.; Sereikaite, J. New β-Carotene-Chitooligosaccharides Complexes for Food Fortification: Stability Study. *Foods*. **2020**, *9* (6), 765 DOI: 10.3390/foods9060765.

4. Ochoa Becerra, M.; Mojica Contreras, L.; Hsieh Lo, M.; Mateos Díaz, J.; Castillo Herrera, G. Lutein as a Functional Food Ingredient: Stability and Bioavailability. *J Funct. Foods*. **2020**, *66*, 103771, DOI: 10.1016/j.jff.2019.103771.
